# Genetic control of pear rootstock-induced dwarfing and precocity is linked to a chromosomal region syntenic to the apple *Dw1* loci

**DOI:** 10.1186/s12870-015-0620-4

**Published:** 2015-09-22

**Authors:** Mareike Knäbel, Adam P. Friend, John W. Palmer, Robert Diack, Claudia Wiedow, Peter Alspach, Cecilia Deng, Susan E. Gardiner, D. Stuart Tustin, Robert Schaffer, Toshi Foster, David Chagné

**Affiliations:** The New Zealand Institute for Plant & Food Research Limited (Plant & Food Research), Fitzherbert Science Centre, Batchelar Road, Palmerston North, 4474 New Zealand; School of Biological Sciences, University of Auckland, Thomas Building 110, 3a Symonds Street, Auckland Central, 1010 New Zealand; Plant & Food Research, Mount Albert Research Centre, 120 Mt Albert Road, Sandringham, Auckland 1025 New Zealand; Plant & Food Research, Hawke’s Bay Research Centre, Cnr Crosses and St George’s Roads, Havelock North, 4130 New Zealand; Plant & Food Research, Motueka Research Centre, 55 Old Mill Road, RD3, Motueka, 7198 New Zealand

**Keywords:** Genetic mapping, Maloideae, *Malus* x *domestica* Borkh, Marker assisted selection, *Pyrus communis* L, SNP, Vigour control

## Abstract

**Background:**

The vigour and precocity of trees highly influences their efficiency in commercial production. In apple, dwarfing rootstocks allow high-density plantings while their precocious flowering enables earlier fruit production. Currently, there is a lack of pear (*Pyrus communis* L.) rootstocks that are equivalent to the high yielding apple rootstock ‘M9’. For the efficient breeding of new *Pyrus* rootstocks it is crucial to understand the genetic determinants of vigour control and precocity. In this study we used quantitative trait loci (QTLs) analysis to identify genetic loci associated with the desired traits, using a segregating population of 405 F1 *P. communis* seedlings from a cross between ‘Old Home’ and ‘Louise Bonne de Jersey’ (OHxLBJ). The seedlings were grafted as rootstocks with ‘Doyenne du Comice’ scions and comprehensively phenotyped over four growing seasons for traits related to tree architecture and flowering, in order to describe the growth of the scions.

**Results:**

A high density single nucleotide polymorphism (SNP)-based genetic map comprising 597 polymorphic pear and 113 apple markers enabled the detection of QTLs influencing expression of scion vigour and precocity located on linkage groups (LG)5 and LG6 of ‘Old Home’. The LG5 QTL maps to a position that is syntenic to the apple ‘Malling 9’ (‘M9’) *Dw1* locus at the upper end of LG5. An allele of a simple sequence repeat (SSR) associated with apple *Dw1* segregated with dwarfing and precocity in pear and was identified in other pear germplasm accessions. The orthology of the vigour-controlling LG5 QTL between apple and pear raises the possibility that the dwarfing locus *Dw1* arose before the divergence of apple and pear, and might therefore be present in other Rosaceae species.

**Conclusion:**

We report the first QTLs associated with vigour control and flowering traits in pear rootstocks. Orthologous loci were found to control scion growth and precocity in apple and pear rootstocks. The application of our results may assist in the breeding process of a pear rootstock that confers both vigour control and precocity to the grafted scion cultivar.

**Electronic supplementary material:**

The online version of this article (doi:10.1186/s12870-015-0620-4) contains supplementary material, which is available to authorized users.

## Background

Commercial apple (*Malus* x *domestica* Borkh.) production relies on the use of dwarfing rootstocks to reduce scion vigour and promote early flowering in young trees [1, 2]. However, the closely related pear (*Pyrus communis* L.) lacks comparable dwarfing *Pyrus* rootstocks, which makes the cultivation of pear currently less profitable than apple. To develop a series of pear rootstocks, it is necessary first to develop an understanding of the mechanisms involved in vigour control and precocity in pear and the genetic determinants of the desired traits.

The physiology of rootstock-induced dwarfing in fruit trees is not fully understood and a number of mechanisms have been suggested to influence dwarfing in perennial fruit tree crops in general. Yonemoto et al. [3] observed that mandarin scions grafted onto rootstocks had a lower sap flow rate and higher soluble solid content than non-grafted trees and Basile et al. [4] found that the daily extension growth of shoots of a peach scion grafted on a semi-dwarfing rootstock was related to the dynamics of stem water potential. In citrus, Lliso et al. [5] found significantly higher concentrations of carbohydrates in fruit and roots of trees on dwarfing rootstocks than on more vigorous ones, suggesting that dwarfing rootstocks promote heavier flowering and crop load and thereby reduce vegetative growth. In apple, research has focused on water and nutrient restriction at the graft union, as well as a reduction of auxin movement from the scion to the rootstock [2, 6–10]. Foster et al. [11] observed that key flowering genes from the *Flowering Time *(*FT*) locus family were up-regulated in dwarfing rootstocks, which would promote flowering and reduce shoot extension growth.

They also found several stress response genes were up-regulated and concluded that stress might be a factor in the dwarfing effect. Recently, two major QTLs (*Dw1 *and *Dw2*), which control most of the dwarfing effect conferred to the scion, have been identified in the apple rootstock ‘Malling 9’ (‘M9’) on LG5 and LG11 respectively, [11–14]. This ‘M9’ dwarfing effect involves the reduction of the number and length of branches in the first year of growth after grafting and an increase in the proportion of floral buds [11, 15]. However, in pear no QTL has been identified that controls tree productivity traits and no genetic analysis has been carried out on rootstocks, although several QTLs have been identified that control traits such as pest and disease resistance [16–18], leaf morphology [19], and fruit quality traits [20–22].

As pear and apple are closely related species within the Rosaceae family [23], and their genomes exhibit a high degree of synteny [24–27], we hypothesized that orthologous loci might occur in both pear and apple that are responsible for the control of scion growth conferred by rootstocks. In the present study, we tested this hypothesis using a segregating population of 405 seedlings from a *P. communis* ‘Old Home’ x ‘Louise Bonne de Jersey’ cross grafted with ‘Doyenne du Comice’ (‘Comice’) scions and phenotyped for precocity and scion growth (vigour). We present the results for a QTL analysis of these traits using a high density genetic map based on single nucleotide polymorphism (SNP) markers anchored to the ‘Bartlett’ v1.0 European pear genome assembly [27].

## Methods

### Segregating population

A cross was made between *Pyrus communis* L. ‘Old Home’ and ‘Louise Bonne de Jersey’ (OHxLBJ), resulting in a segregating population consisting of 421 F1 seedlings. The seedlings were grown in the glasshouse for three months and planted out into the Plant & Food Research orchard in Motueka, New Zealand (41°6’S; 172°58’E). After 2 months of acclimatisation, the seedlings were summer budded with ‘Comice’ (*Pyrus communis* L.). In the following spring when the trees were cut down to the bud, grafts from the shoots removed from the OHxLBJ seedlings were inserted onto *Pyrus calleryana* seedling rootstocks to provide leaf material for DNA extraction. As controls, fifty clonal *Cydonia oblonga* ‘Quince C’ (QC) rootstocks grafted with ‘Comice’ were systematically distributed throughout the orchard block to give some indication of the variation in growing conditions across the block. The trees were planted into three rows, each containing 157 trees, including the QC controls, with a spacing of 0.8 m within the row and 3.3 m between the rows. Of the original 421 seedlings, propagation of scions failed on 16 trees, leaving 405 for phenotyping. To avoid any horticultural influence on tree shape and vigour, the trees were neither pruned nor trained. Once the trees began to flower and crop, all fruit were removed from the trees each season after first drop to avoid biennial bearing, bending of the branches (to prevent increasing precocity) and a confounding effect of the crop on tree vigour. Drip irrigation, fertilisation and pest and disease control were carried out; woven plastic mat was laid down to repress weed growth.

### Architectural measurements and inflorescence assessment

Scions were phenotyped for architectural traits for the first four years of growth after grafting (years 1–4) (Table 1). Detailed architectural measurements were taken after growth cessation (June/July) in years 1–3, including trunk cross-sectional area (TCA) 20 cm above the graft union; length of main axis (length taken for each new growing cycle); and number of branches and spurs (short shoots <2.5 cm) (Table 1). Branches were classified as either sylleptic shoots, which extend in the same year they are initiated, or proleptic shoots, which extend after winter dormancy [28].

**Table 1:** Architectural measurements taken over the first four years of growth after grafting

Trait	Year 1	Year 2	Year 3	Year 4	Year 5
Number of branches per tree (*Branches*)	x	x	x		
Total tree height (*Height*)	x	x	x	x	
Length of the new main axis growth (*LNG*)	x	x	x	x	
Number of inflorescence (*Inflorescence*)			x	x	x
Number of nodes per tree (*Nodes*)	x	x			
Number of spurs per tree (*Spurs*)	x	x	x		
TCA 20 cm above graft unit (*TCAtrunk*)	x	x	x	x	
TCA of the rootstock (*TCAroot*)			x	x	
TCA secondary growth of the main axis (*TCAsec*)			x		
TCA tertiary growth of the main axis (*TCAtert*)			x		
Vigour classification (*Size*)			x		
Root suckering (*Suckers*)			x		

Measurements were taken for OHxLBJ pear rootstock segregating population and Quince C (QC) controls grafted with scions of ‘Comice’. TCA trunk cross-sectional area, spurs are short shoots (<2.5 cm). The designation for the variables used for QTL analysis is indicated between brackets

**Fig. 1:**
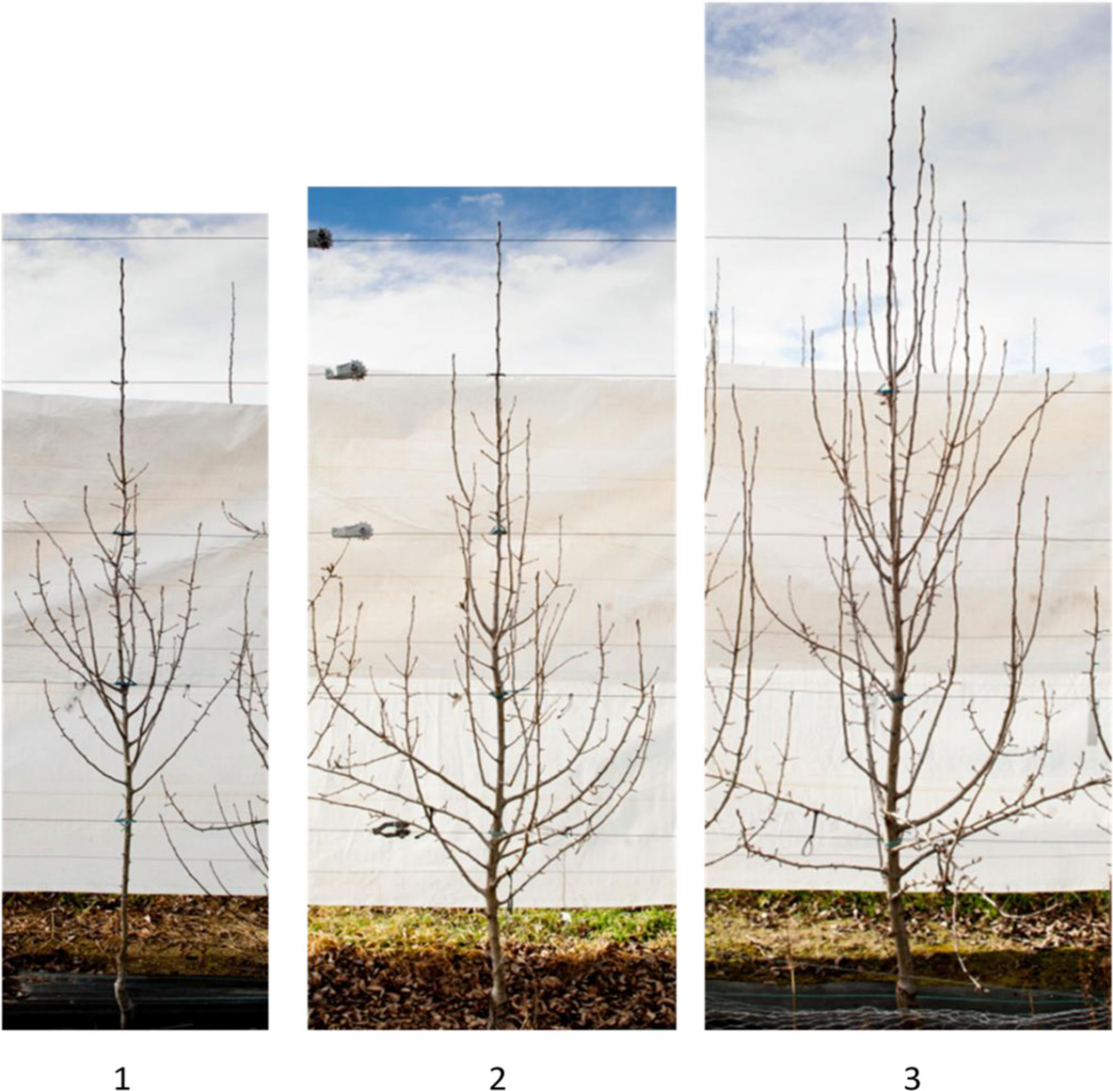
Examples of three vigour classes of trees in the second year of growth after grafting. Trees shown are 1) small, 2) moderate, 3) vigorous ‘Old Home’ x ‘Louise Bonne de Jersey’ pear rootstocks grafted with ‘Comice’. The wires indicate the height of the trees, with the wire being 0.8, 1.3, 1.8, 2.3, 2.75 m from the ground, lowest to the top respectively

In year 3, the tree canopies were visually categorised as being small, moderate or vigorous, using QC controls as models for moderate tree growth. An example for the three vigour classifications can be seen in Fig. 1. The presence or absence of root suckers was recorded in the third year. The first inflorescence assessment was done at the beginning of year 3 and repeated in the following two springs. No ‘Comice’ scions flowered either on the seedlings or on QC control rootstocks in year 2. In year 3, the total number of inflorescences was counted and their positions recorded; this was repeated in year 4. At the beginning of year 5, the proportions of inflorescence production were estimated according to the size of the tree, relative to the tree with the highest number of inflorescences. The trees were ranked into classes from 0–4, with 0 = no flowers, 1 = 1–25 %, 2 = 26–50 %, 3 = 51–75 %, 4 = 76–100 %.

### Data analysis

Univariate mixed models were fitted to the data with row and a linear effect of tree position in the row as fixed effects, and genotype as the only random effect. Localized spatial trends were modelled by fitting first-order auto-correlations for tree positions [29]. The fixed effects were chosen based on an examination of the variograms when fitting the first-order auto-correlations to both row and tree position, and the auto-correlations to retain were based on likelihood ratio tests. Having determined the optimal univariate model, it was then extended to bivariate models for every pairwise set of variates. These bivariate models allowed for separate fixed and spatial effects for each variate, and also a different genetic variance for each, as well as the genetic correlation. Predicted values from these bivariate analyses were used in the QTL analysis. Data from each year were analysed separately, in order to check whether the putative QTLs were stable across years. Residual plots were examined to check for outliers and assess the validity of the normality assumption. For all variates apart from *Branches_year2*, a square-root transformation was used to obtain a satisfactory approximation to normality. Basic statistical analysis was carried out using Minitab 16 Statistical Software (2010 Minitab Inc.). All further analysis were conducted using R 3.0.1 [30], and the mixed models were fitted using the asreml package version 3.0-1 [31].

### Genetic mapping and QTL analysis

DNA was extracted using a CTAB method [32], followed by purification with NucleoSpin® columns (Macherey-Nagel GmbH & Co. KG). DNA was quantified using a NanoDrop™ 2000c spectrophotometer (Thermo Fisher Scientific Inc.). SNP marker genotyping was performed using the apple and pear Infinium® II IRSC 9 K SNP array [33, 34] on 297 segregating individuals and both parents. Genomic DNA was amplified and hybridized to the apple and pear Infinium® II IRSC 9 K SNP array following the Infinium® HD Assay Ultra protocol (Illumina Inc., San Diego, USA) and scanned with the Illumina HiScan. Data were analysed using Illumina’s GenomeStudio v 1.0 software and genetic mapping carried out using JoinMap 3® [35]. A LOD score of 5 or higher was used for grouping and the genetic distance within the group was calculated using the Kosambi function. The linkage groups (LGs) were identified by aligning the parental maps of OH and LBJ to the map developed by Montanari et al. (2013), which contains apple and pear SSR markers from the ‘Bartlett’ consensus map of Celton et al. [24]. The map was drawn and aligned using MapChart v.2.2 [36]. The parental genetic maps were used with raw and transformed phenotypic data of tree growth, precocity and suckering for QTL analysis employing MapQTL5 [37]. For normally distributed data, Interval Mapping (IM) followed by Multiple QTL Mapping (MQM) was performed and a permutation test (1000 permutations) was used to calculate the LOD threshold for QTL significance. ANOVA was used to calculate the percentage of the phenotypic variance explained by each QTL. When normalisation of the data failed, the Kruskal-Wallis test was used for QTL detection.

### Identification of the dwarfing allelotype in a pear germplasm selection

The SSR marker Hi01c04, developed by Silfverberg-Dilworth et al. [38] and identified as the proximal flanking marker for the Dw1 region on LG5 of apple [12] was screened over 96 individuals of the OHxLBJ population to determine the linkage phase between the QTL and the SSR alleles. PCR amplification was carried out using a modified version of the fluorescent M13 universal primer system [39] and a touchdown PCR programme with annealing temperature 60–55 °C (94 °C/2 min 45 s; 10 cycles: 94 °C/55 s, 60 °C/55 s (−0.5 °C per cycle); 72 °C/1 min 30 s; 30 cycles: 94 °C/55 s, 55 °C/55 s, 72 °C/1 min 30 s; 72 °C/10 min). The fragments were separated using the ABI3500 sequencer, and their size analysed with GeneMarker® v 2.2.0 software (© SoftGenetics, LLC.). The marker was then included in the OH map. The allele sizes were compared with those detected by screening the same SSR marker over 92 pear accessions from selections of germplasm from France, New Zealand, Germany and the USA, including OH and LBJ.

### Finding orthologous loci in pear and apple

Apple and European pear regions were compared to identify orthologous genes using OrthoMcl2.0.3. [40]. Synteny gene blocks were detected with OrthoCluster [41]. *Pyrus* scaffolds were aligned to *Malus* scaffolds using the MUMmer 3.3 package [42]. Pear scaffolds were further filtered based on having at least two alignments, each alignment longer than 2kbp or total alignment length not shorter than 3kbp.

## Results

### Architectural measurements

Architectural measurements were taken on ‘Comice’ scions grafted on both the OHxLBJ segregating population and QC controls from the first to the fourth years of growth (Table 1). The phenotypic variability of the raw data is illustrated in Table 2. A wide range of vigour was observed in the grafted scions as early as in the first year of growth. In total, 343 trees (89 %) of the OHxLBJ population developed sylleptic shoots in year 1, of which 87 trees (25 %) grew more than 10 sylleptic shoots.

**Table 2:** Phenotypic variability for scion architecture and flowering

Variable	Year		N	Mean	SE Mean	StDev	Min	Q1	Median	Q3	Max
Number of branches per tree	1	OHxLBJ	385	6.8	0.2	4.6	0	3	7	10	20
		QC	49	8.4	0.7	4.9	0	5	8	11	21
	2	OHxLBJ	389	37.5	0.8	15.9	0	26	38	47	107
		QC	50	36.2	2.1	14.6	10	25	37	47	74
	3	OHxLBJ	276	60.4	2.0	32.6	0	38	59	81	169
		QC	49	42.7	3.3	23.3	7	27	39	58	108
Total tree height	1	OHxLBJ	382	127.5	1.3	25.0	14	121	134	142	183
		QC	47	110.5	3.7	25.3	52	101	118	126	157
	2	OHxLBJ	382	205.5	1.7	33.5	65	188	203	226	303
		QC	47	191.5	4.3	29.5	129	168	190	206	251
	3	OHxLBJ	379	298.4	2.6	50.4	74	270	302	332	443
		QC	46	284.8	5.7	38.4	181	260	285	318	354
	4	OHxLBJ	403	376.8	4.6	91.7	29	350	400	433	546
		QC	50	347.4	13.0	91.9	58	330	374	400	449
Length of the new main axis growth	2	OHxLBJ	388	78.0	1.2	24.5	10	60	69	97	154
		QC	50	82.1	2.5	17.8	47	70	81	91	121
	3	OHxLBJ	402	93.3	1.4	27.9	6	75	99	114	144
		QC	49	93.2	3.1	21.5	52	77	96	112	127
	4	OHxLBJ	400	96.9	0.9	18.5	11	92	101	108	127
		QC	47	90.9	2.7	18.6	18	82	94	105	116
Inflorescence	3	OHxLBJ	405	12.5	1.1	23.0	0	0	2	15	136
		QC	50	45.9	6.1	43.1	0	10	35	67	176
	4	OHxLBJ	403	117.9	4.3	85.6	0	44	110	181	458
		QC	50	119.6	8.9	62.7	0	71	114	161	293
Axillary inflorescence	2	OHxLBJ	405	2.2	0.4	7.1	0	0	0	0	63
		QC	50	22.5	4.1	28.8	0	2	11	36	123
	4	OHxLBJ	402	4.3	0.4	8.5	0	0	1	5	69
		QC	49	5.9	0.9	6.0	0	1	4	11	22
Number of nodes per tree	1	OHxLBJ	383	42.6	0.4	7.1	8	40	44	47	58
		QC	47	38.0	1.0	7.1	18	34	38	43	50
	2	OHxLBJ	386	32.6	0.4	7.4	9	27	30	40	50
		QC	50	32.8	0.8	5.8	22	28	32	38	42
Number of spurs per tree	1	OHxLBJ	385	10.1	0.3	6.8	0	5	9	14	42
		QC	49	5.6	0.6	3.9	0	3	5	9	16
	2	OHxLBJ	389	43.3	1.7	33.0	0	15	36	66	179
		QC	50	42.7	4.4	31.2	0	16	39	57	124
	3	OHxLBJ	276	197.5	5.0	83.5	15	134	195	253	557
		QC	49	242.8	14.2	99.7	56	159	238	315	454
TCA of the trunk	1	OHxLBJ	383	0.6	0.0	0.2	0	0	1	1	1
		QC	47	0.7	0.0	0.3	0	1	1	1	1
	2	OHxLBJ	387	1.7	0.0	0.7	0	1	2	2	5
		QC	50	1.9	0.1	0.6	1	1	2	2	3
	3	OHxLBJ	404	3.8	0.1	1.6	0	3	4	5	10
		QC	50	4.0	0.2	1.2	2	3	4	5	7
	4	OHxLBJ	402	5.9	0.1	2.6	0	4	6	8	14
		QC	49	5.4	0.2	1.6	2	4	5	7	10
TCA of the rootstock	3	OHxLBJ	404	7.5	0.1	2.8	1	6	7	9	18
		QC	50	3.3	0.1	1.0	2	3	3	4	6
	4	OHxLBJ	402	10.6	0.2	4.1	1	8	10	13	27
		QC	49	5.1	0.2	1.7	2	4	5	6	11
TCA secondary main axis growth	3	OHxLBJ	399	1.1	0.0	0.5	0	1	1	1	4
		QC	49	1.3	0.1	0.4	1	1	1	2	2
TCA tertiary main axis growth	3	OHxLBJ	399	0.6	0.0	0.3	0	0	1	1	3
		QC	49	0.7	0.0	0.2	0	1	1	1	1

Variability is shown for the pear OHxLBJ segregating population and Quince C (*QC*) controls grafted with ‘Comice’. *TCA* Trunk cross-sectional area, *N* Number of non-missing values, *SE Mean* Standard error of mean, *StDev* Standard deviation, *Q1* First quartile, *Q3* Third quartile

After proleptic shoots developed in the second year of growth, a large variability was observed in the total number of branches, with a range of zero to 107 branches per tree. After the third year, third-order branches and spurs grew off the second-order sylleptic and proleptic branches. This branching habit was repeated in the following growing cycle, resulting in a very complex tree structure which could be ranked into three vigour classes based on overall tree size, with 55 small, 200 moderate and 148 vigorous phenotypes (Fig. 1).

Flowering first occurred at the beginning of the third year of growth after grafting for 257 individuals (63 %) of the OHxLBJ population. The following spring (year 4), 398 trees flowered. In year 5, 56 of the trees (14 %) did not flower, of which only five (1 %) had never flowered before. Flowering occurred mainly on spurs and terminal buds, with an average of 10.5 flower clusters per tree in year 3 and 113.6 in year 4 for the OHxLBJ population. High numbers of axillary (1-year-old lateral bud) flower clusters were found on the scions grafted onto the QC controls in year 3, with an average of 22.5 axillary buds and 116 spurs and terminal buds per tree. The trees grafted onto OHxLBJ showed only minimal axillary flowering in year 3 and year 4, with averages of 2.2 and 4.3 respectively. In total, 247 rootstocks exhibited root suckering, while 161 did not. Root suckering was detected for 38 (69 %) out of 55 of the trees classified as small, 128 (64 %) out of 199 moderate trees, and 77 (52 %) out of 148 vigorous trees. Trees with root suckering had a significantly smaller average TCA than those without (3.65 cm^2^ and 4.28 cm^2^, respectively; *p* = 0.002).

### Correlation between traits

**Fig. 2:**
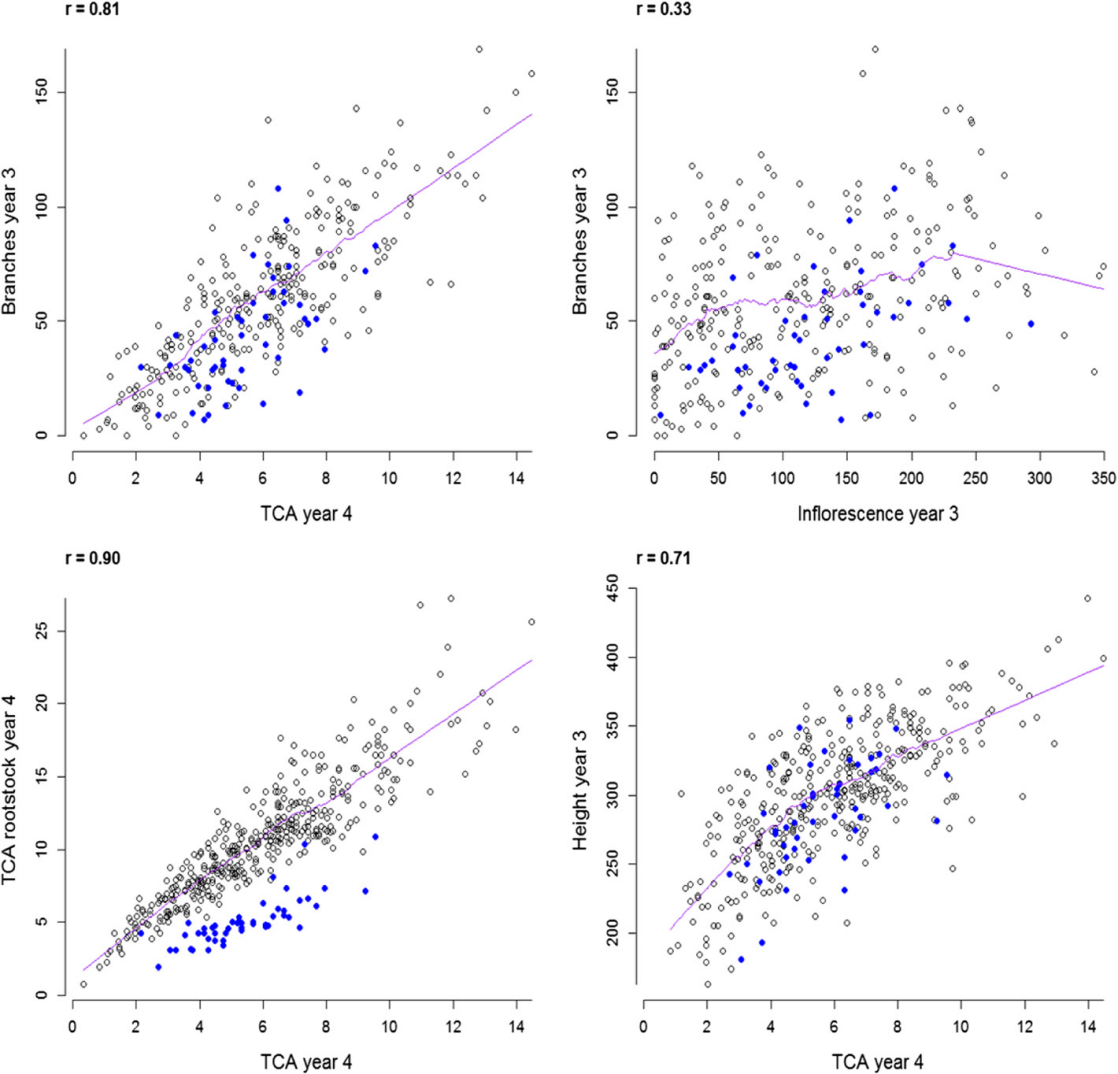
Scatterplots between different pear architectural and flower traits designed with RStudio. TCA: trunk cross-sectional area. Black circles represent ‘Old Home’ x ‘Louise Bonne de Jersey’ (OHxLBJ) seedlings and blue dots ‘Quince C’ controls. The purple line shows a “Friedman's super smoother” (span = 0.2). The correlation coefficients (shown at the top left of each plot) were calculated for the OHxLBJ values only

The raw phenotypic data were used to look at relationships between traits. Figure 2 shows some selected correlation graphs, while the correlation matrix for all traits, with Pearson correlations (r) and their significance values, can be found in Additional file 1: Table S1. A significant positive correlation (*r* = 0.81) was observed between *Branches_year3* and the *TCAtrunk_year4 *and between the *Height_year3 *and the *TCAtrunk_year4 *(*r* = 0.71). As expected, the highest correlation (*r* = 0.9) was found between the *TCAtrunk_year4 *and the *TCAroot_year4*, showing the consistency in the measurements.

**Fig. 3:**
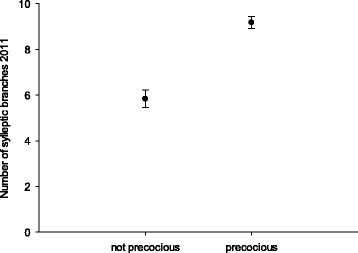
Interval plot of first-year (2011) sylleptic branching comparing precocious and non precocious trees. Symbols show the mean (precocious = 9.2; not precocious = 5.8) and the error bars of the mean (precocious = 0.38; not precocious = 0.27) (p-value = 0.000)

**Fig. 4:**
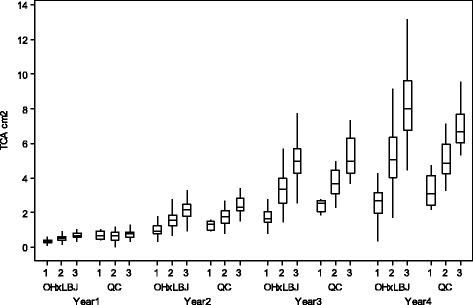
Box-plots of each year’s trunk cross-sectional area (TCA) comparing three vigour classes. 1) small, 2) moderate, 3) vigorous within the ‘Old Home’ x ‘Louise Bonne de Jersey’ (OHxLBJ) pear population and the ‘Quince C’ (QC) controls. Box-plot symbols show the median, Q1 and Q3, and the highest and lowest values

No strong positive correlation between flowering and architectural traits was found. However, trees that flowered early (year 3) had significantly more sylleptic branches than those that did not (Chi-square = 31.49, p-value = <0.0005) (Fig. 3). The TCA showed the strongest correlation with other traits and was therefore a representative measurement for tree vigour, becoming a stronger indicator for overall tree size with each annual growth cycle (Fig. 4). The variation in vigour of the scions budded onto the QC rootstocks indicates the environmental influence within the orchard.

### Analysis of the phenotypic variability within the orchard and among genotypes

Positional effects within the orchard were accounted for by using three different linear mixed models: Model 1: first-order autocorrelation for both row and plant position within the row; Model 2: first-order autocorrelation for only the plant position; Model 3: no autocorrelation for both row and plant position. For *Branches_year1-3*, *Height_year1 + 3*, *Inflorescence_year2 + 3*, *Spurs_year2 + 3*, *TCA_year3 + 4*, *TCAroot_year3 + 4* and *TCAsec_year3*, the row and plant position auto-correlation did not improve the fit. For the *Height_year4*, *LNG_year2-4*, *Nodes_year2*, *Spurs_year1*, *TCA_year2 *and the *TCAtert_year3*, the plant position improved the fit and Model 2 was used for bivariate models for QTL detection. The clonal QC controls should arguably be fitted as fixed effects. This was tested with a few key variates and the breeding values obtained were very similar to those obtained from the model described. Square root transformation was necessary to normalise the data for all traits recorded, except for *Branches_year2*. However, some variables (*LNG_year2 + 3*, *Height_year1 + 2 + 3*, *Nodes_year2*, *Spurs_year1 + 3*, *TCA_year2 *and the *Inflorescence_year2 + 4*) showed marked deviations from normality, even after transformation.

### Genetic Map construction

High density genetic maps were constructed for both parents using 597 and 113 polymorphic pear and apple SNP markers [33] respectively (Table 3). The OH map consists of 17 linkage groups (LG) representing the 17 chromosomes of the pear genome. Only 16 linkage groups were obtained for LBJ, with LG17 being absent.

**Table 3:** Number of pear and apple markers in ‘Old Home’ (OH) and ‘Louise Bonne de Jersey’ (LBJ) genetic maps

	Apple	Pear	total	LGs	cM
OH	58	341	399	17	913
LBJ	64	382	446	16	1044
Common	9	126	135		

*LGs* number of linkage groups, *cM* total length of the genetic map in centiMorgans

The genetic maps of OH and LBJ were aligned with parental maps of ‘Moonglow’ (Moon) and PEAR1 [33] which contain SSR markers derived from apple (Additional file 2: Figure S1).

### QTL detection

QTLs were detected using the OH and LBJ parental genetic maps for the tree architecture and flowering traits across four years (Tables 4 and 5, Additional file 2: Figure S1). Significant QTLs for the control of the number of branches were detected in three successive years on LG5 and LG6 of OH. A small-effect QTL controlling *Branches_year1 *was located on LG6 of LBJ and was also detected in year 3. In the first year of growth after grafting, significant QTLs were detected for the *TCAtrunk_year1 *on LG16 and 6 of OH. The LG6 QTL was confirmed in years 2, 3 and 4, whereas the LG16 QTL was not reproducible. A QTL influencing *TCAtrunk* was detected on LG5 of OH in both years 3 and 4. Additional smaller-effect QTLs controlling *TCAtrunk*, inherited from LBJ, were detected on LG13 and LG6. QTLs influencing *LNG* were detected on OH LG5 in years 2–4 and these co-located with the *TCAtrunk* QTL. Smaller-effect QTLs controlling the *LNG* from LBJ were located on LG6 and LG7; however, only the LG6 QTL could be replicated across two years. QTLs controlling the *TCAsec_year3 *and *TCAtert_year3 *(only measured in year 3), *Height* and the *Spurs* per tree were detected on LG5 and LG6 of OH and LG6 and LG1 of LBJ. A QTL controlling *Size_year3 *was detected on LG5 and LG6 of OH, co-locating with the *TCAtrunk* and *Height *QTLs. The architectural OH LG5 QTLs explained between 5.44 % and 16.6 % of the variability for *Spurs_year2 *and *TCAsec_year3*, respectively. The variance explained for the OH LG6 QTLs ranked from 3.98 % for *TCAtrunk_year3 *to 16.42 % for *Height_year3*. The highest variance explained by any LBJ LG6 QTL was 7.72 % for the QTL controlling the *TCAtert_year3*, and the lowest was 4.25 % for the QTL influencing *Branches_year1*. A QTL controlling *Inflorescence* phenotyped at the beginning of the third year after grafting was detected on LG5 of OH, co-locating with the tree architecture QTLs. No flowering-related QTLs were detected segregating from LBJ. A QTL controlling *Suckers_year3 *was detected on LG5 of OH.

**Table 4:** QTLs detected for pear architectural and precocity traits for predicted and normalised data

Parent	LG	Traits	Marker with highest LOD	Marker position (cM)	LOD		% Expl.
OH	5	*Branches_year1*	ss475878191	2.2	6.70	^c^	10.01
OH	5	*Branches_year2*	ss475878191	2.2	3.53	^c^	5.48
OH	5	*Inflorescence_year4*	ss475878191	2.2	13.06	^c^	18.31
OH	5	*LNG_year4*	ss475878191	2.2	11.13	^c^	16.16
OH	5	*Spurs_ year2*	ss475878191	2.2	3.51	^c^	5.44
OH	5	*TCAtrunk_year3*	ss475878191	2.2	12.37	^c^	15.99
OH	5	*TCAtrunk_ year4*	ss475878191	2.2	11.31	^c^	16.42
OH	5	*Branches_ year3*	ss527788221	1.6	6.74	^c^	11.16
OH	5	*Height_ year3*	ss527789077	0.0	8.57	^c^	12.69
OH	5	*Nodes_ year1*	ss527789704	1.1	5.67	^c^	9.54
OH	5	*TCAroot_ year4*	ss527789704	1.1	7.89	^c^	12.45
OH	5	*TCAsec_ year3*	ss527789704	1.1	10.97	^c^	16.6
OH	5	*TCAtert_ year3*	ss527789704	1.1	10.61	^c^	15.23
OH	5	*TCAroot_ year3*	ss527789704	1.1	5.55	^c^	8.97
OH	6	*Height_ year3*	ss475883025	5.2	4.37	^c^	16.42
OH	6	*Branches_ year1*	ss527789305	6.5	5.29	^c^	7.79
OH	6	*Branches_ year2*	ss527789305	6.5	5.04	^c^	7.93
OH	6	*Branches_ year3*	ss527789305	6.5	4.93	^c^	7.24
OH	6	*Nodes_ year1*	ss527789305	6.5	2.95	^b^	4.72
OH	6	*Spurs_ year2*	ss527789305	6.5	5.00	^c^	7.48
OH	6	*TCAroot_ year4*	ss527789305	6.5	3.21	^c^	4.81
OH	6	*TCAsec_ year3*	ss527789305	6.5	4.37	^c^	6.25
OH	6	*TCAtert_ year3*	ss527789305	6.5	3.46	^c^	6.46
OH	6	*TCAtrunk_ year1*	ss527789305	6.5	5.08	^c^	7.3
OH	6	*TCAtrunk_ year3*	ss527789305	6.5	3.46	^c^	3.98
OH	6	*TCAtrunk_ year4*	ss527789305	6.5	4.66	^c^	6.71
OH	6	*TCAroots_ year3*	ss527789305	6.5	3.51	^c^	5.28
LBJ	6	*Branches_ year1*	ss475878560	47.8	2.58	^a^	4.25
LBJ	6	*Spurs_ year2*	ss475878560	47.8	2.78	^a^	4.68
LBJ	6	*TCAtrunk_ year4*	ss475878560	47.8	3.72	^b^	6
LBJ	6	*Branches_ year3*	ss527787860	60.6	2.75	^a^	5.27
LBJ	6	*Height_ year3*	ss527787915	59.4	2.77	^a^	5.65
LBJ	6	*TCAsec_ year3*	ss527789084	21	4.14	^c^	6.33
LBJ	6	*TCAtert_ year3*	ss527789084	21	5.05	^c^	7.72
LBJ	7	*TCAtert_ year3*	ss527789229	31.0	3.25	^b^	4.79
LBJ	16	*TCAroot_ year4*	ss527788231	56.2	3.02	^b^	4.2

QTLs were detected coming from ‘Old Home’ (OH) and ‘Louise Bonne de Jersey’ (LBJ). Percentage of the phenotypic variance explained by each QTL (% Expl.) was calculated using ANOVA. See Table 1 for an explanation of the variables. LOD score indicates the genome-wide significance of the QTL ^a^: 90 %, ^b^: 95 % and ^c^: 99 %

**Table 5:** QTLs detected for pear architectural and precocity traits for predicted (bivariate analysis), non-normally distributed data

Parent	LG	Trait	Marker with highest LOD	Marker position (cM)	K value	P-value
OH	5	*Size_year3*	ss475878225	4.0	24.6	0.0001
OH	5	*Height_year1*	ss527788221	1.6	25.1	0.0001
OH	5	*Height_year2*	ss527788221	1.6	32.2	0.0001
OH	5	*Suckers_year3*	ss527788221	1.6	20.9	0.0001
OH	5	*Inflorescence_year3*	ss527789278	0.7	46.6	0.0001
OH	5	*Height_year4*	ss527789704	1.1	37.4	0.0001
OH	5	*Inflorescence_year5*	ss527789704	1.1	60.8	0.0001
OH	5	*LNG_year2*	ss527789704	1.1	22.2	0.0001
OH	5	*LNG_year3*	ss527789704	1.1	47.7	0.0001
OH	5	*Nodes_year2*	ss527789704	1.1	22.0	0.0001
OH	5	*Spurs_year3*	ss527789704	1.1	38.4	0.0001
OH	6	*Height_year1*	ss527789305	49.0	19.2	0.0001
OH	6	*Height_year2*	ss527789305	49.0	17.6	0.0001
OH	6	*Height_year4*	ss527789305	49.0	16.4	0.0001
OH	6	*Size_year3*	ss527789305	49.0	16.1	0.0001
LBJ	1	*Height_year2*	ss475876925	54.0	11.5	0.001
LBJ	1	*Height_year4*	ss527789822	32.1	9.4	0.005
LBJ	6	*TCA_year2*	ss475876015	37.3	12.5	0.0005
LBJ	6	*Height_year4*	ss475878560	12.8	13.0	0.0005
LBJ	6	*LNG_year2*	ss527787800	41.7	16.2	0.0001
LBJ	6	*Spurs_year3*	ss527787800	41.7	17.6	0.0001
LBJ	6	*Height_year2*	ss527787860	0.0	12.0	0.001
LBJ	6	*LNG_year3*	ss527788212	27.8	8.4	0.005
LBJ	6	*Nodes_year2*	ss527788579	41.7	14.0	0.0005
LBJ	6	*Height_year1*	ss527789592	9.3	12.1	0.001
LBJ	7	*LNG_year3*	ss527789852	35.5	13.2	0.0005
LBJ	7	*Suckers_year3*	ss527789852	35.5	11.0	0.001
LBJ	10	*Size_year3*	ss527788181	48.4	14.7	0.0005

Displayed QTLs, derived from ‘Old Home’ (OH) and ‘Louise Bonne de Jersey’ (LBJ), show the closest marker and its position on the linkage group (LG). Significance was calculated using the Kruskal-Wallis (K value) analysis. See Table 1 for an explanation of the variables

### Synteny between apple and pear dwarfing QTLs

**Fig. 5:**
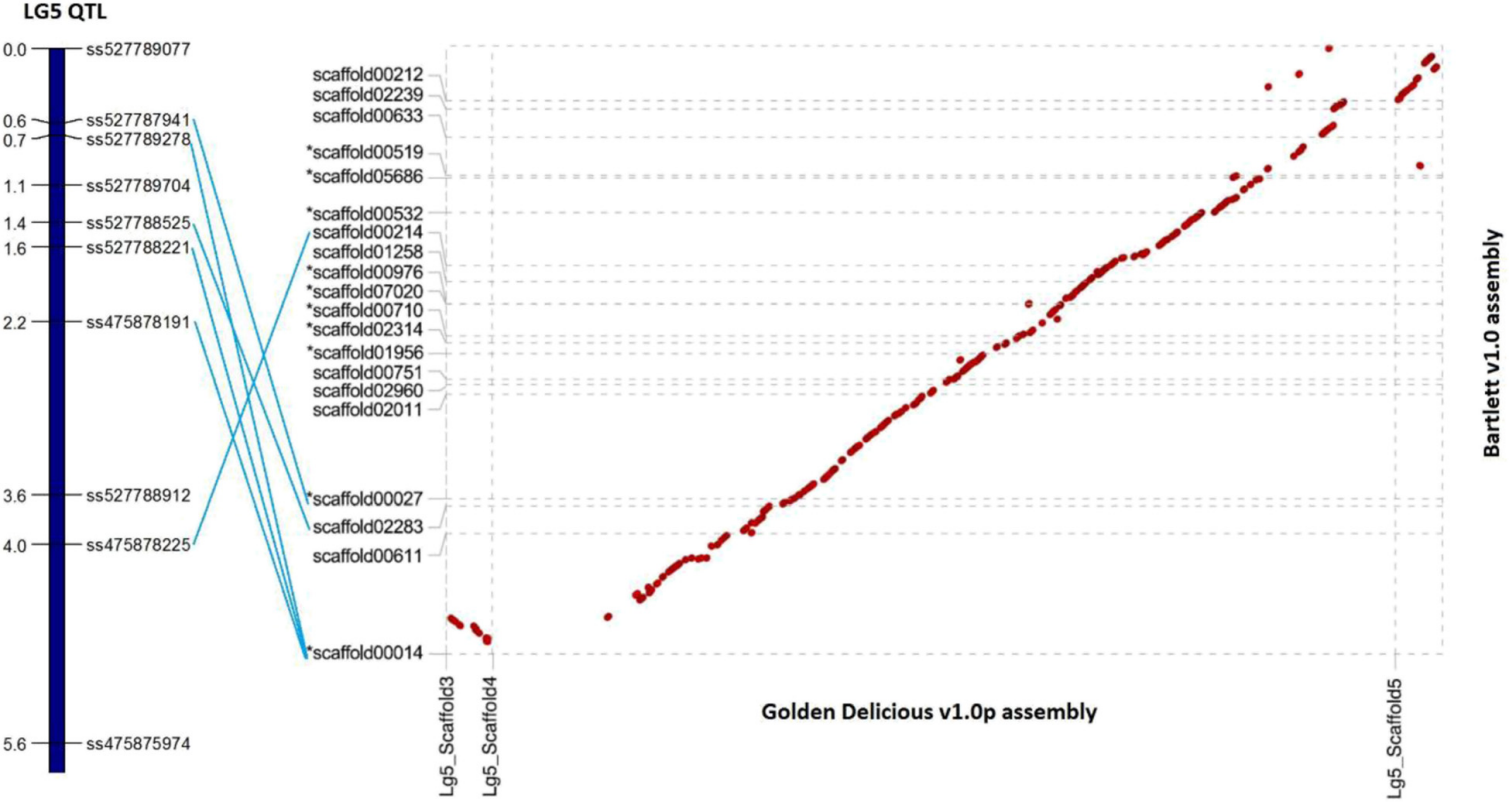
Alignment of the pear (‘Bartlett’ v1.0) and apple (‘Golden Delicious’ v1.0p) genomes in the orthologous QTL region. The Dot-plot shows the alignments (longer than 2kbp and >90 % identity) of the apple (X-axis) and European pear (Y-axis) genomes in the LG5 QTL region controlling vigour and precocity in apple and pear. “*” in front of the scaffold name represents the scaffold is aligned in the opposite direction

Alignment of the top of LG5 of the apple and pear genomes (Fig. 5) showed that the OH LG5 QTL for rootstock control of architecture and flowering traits is syntenic to the dwarfing and precocity *Dw1* QTL detected in apple ‘M9’ rootstocks (Foster et al. 2015). The pear LG5 QTL markers with the highest LOD scores are located on scaffolds 3 and 4 on LG5 of the ’Golden Delicious’ v1.0 genome [43]. After filtering, 20 *Pyrus* scaffolds mapped to three *Malus* scaffolds (Scaffold3, 4 and 5) on LG5. Only alignments longer than 2kbp and with >90 % identity are drawn on Fig. 5. Three of the markers with the highest LOD scores for the total number of flowers (year 3) and the TCA of the trunk (year 3) were located on ‘Bartlett’ v1.0 Scaffold00014, and could be aligned with loci on Scaffold3 and Scaffold4 of the ‘Golden Delicious’ v1.0 LG5. Two other markers mapped to ‘Bartlett’ v1.0 Scaffold00214 and Scaffold00116, and were both aligned to Scaffold4 in ‘Golden Delicious’ v1.0 LG5. ‘Golden Delicious’ Scaffold3, 4 and 5 span approximately 1.33Mbp of the apple genome and the 20 ‘Bartlett’ v1.0 scaffolds cover 3.45Mbp of the European pear genome in total.

### Dwarfing and precocity

Architectural QTLs were mainly detected on LG5 and LG6 of OH. QTLs for the control of the total number of inflorescences co-located with the architectural QTLs on LG5 of OH. The effects of the QTLs indicate that smaller trees tended to have delayed flowering. Analysis of the genotype of the marker with the highest LOD score (ss475878191) of the LG5 QTL (Table 6) demonstrated that individuals carrying the high vigour genotype (AA) had a higher tendency for precocity, with 74 % being precocious, while 50 % of the individuals with the low vigour genotype (AB) were precocious. However, only 14 % of the total population had the desired low vigour and precocious phenotype, with more individuals carrying the AB allelotype.

**Table 6:** Segregation of dwarfing and precocity among the seedlings in the OHxLBJ population using marker ss475878191

Year 3	TCA (cm^2^)	No inflorescence	+ inflorescence
AA	<3	11 (10 %)	12 (11 %)
	3-4.5	10 (9 %)	28 (26 %)
	>4.5	8 (7 %)	41 (37 %)
AB	<3	40 (25 %)	25 (16 %)
	3-4.5	27 (17 %)	34 (22 %)
	>4.5	11 (7 %)	21 (13 %)
Total population	<3	51 (19 %)	37 (14 %)
	3-4.5	37 (14 %)	62 (23 %)
	>4.5	19 (7 %)	62 (23 %)

Low vigour individuals are represented by a trunk cross-sectional area (TCA) smaller than 3 cm^2^ and high vigour have a TCA greater than 4.5 cm^2^. The ss475878191 SNP segregates as ABxAA in the OHxLBJ population

### Detection of the LG5 precocious allele in a pear germplasm set

The microsatellite marker Hi01c04 that was located within the QTL region on LG5 (Additional file 2: Figure S1) was heterozygous in both OH (116 bp and 121 bp alleles) and LBJ (113 bp and 117 bp alleles). The 121 bp fragment was more frequent in precocious OHxLBJ segregating individuals (Table 7).

**Table 7:** Genotyping of the SSR marker Hi01c04 in pear germplasm accessions

Accession				
	Vigour/precocity	Germplasm	Family	Allele size (bp)
‘Old Home’ (OH)		PFR, NZ	*Pyrus communis*	**116**;121
‘Louise Bonne de Jersey’ (LBJ)		PFR, NZ	*Pyrus communis*	113;117
OHxLBJ105	High/ early	PFR, NZ	*Pyrus communis*	117;121
OHxLBJ109	High/ early	PFR, NZ	*Pyrus communis*	113;121
OHxLBJ185	High/ early	PFR, NZ	*Pyrus communis*	113;121
OHxLBJ129	High/ early	PFR, NZ	*Pyrus communis*	117;121
OHxLBJ118	Low/ early	PFR, NZ	*Pyrus communis*	**116**;-
OHxLBJ122	Low/ late	PFR, NZ	*Pyrus communis*	113;**116**
OHxLBJ176	Low/ late	PFR, NZ	*Pyrus communis*	**116**;-
OHxLBJ172	Low/ late	PFR, NZ	*Pyrus communis*	113;**116**
‘Bartlett’		Corvallis, USA	*Pyrus communis*	113;117
‘Beurre Hardy’		PFR, NZ	*Pyrus communis*	113;-
‘Beurre Hardy - Royal Red’		Corvallis, USA	*Pyrus communis*	**116**;-
‘Bishops Thumb’		PFR, NZ	*Pyrus communis*	113;-
‘Bon Chrétien d'Hiver’		Corvallis, USA	*Pyrus communis*	115;-
‘Bosc’		PFR, NZ	*Pyrus communis*	113;-
BP 1		INRA, France	*Pyrus communis*	112;**116**
BP 3		INRA, France	*Pyrus communis*	113;-
‘Brokmal’		Bundessortenamt, Germany	*Pyrus communis*	113;121
BU 2-33		Bundessortenamt, Germany	*Pyrus communis*	113;121
BU 3		Bundessortenamt, Germany	*Pyrus communis*	115;127
‘Comice’		PFR, NZ	*Pyrus communis*	113;124
‘Conference’		PFR, NZ	*Pyrus communis*	113;**116**
‘Crimson Gem’		PFR, NZ	*Pyrus communis*	113;124
‘Farmingdale’		Corvallis, USA	*Pyrus communis*	113;-
‘Fox’		Corvallis, USA	*Pyrus communis*	**116**;121
‘Fox 11’		INRA, France	*Pyrus communis*	113;117
‘Fox 16’		INRA, France	*Pyrus communis*	115;-
‘Joey's Red Flesh Pear’		Corvallis, USA	*Pyrus communis*	115;-
‘Le Nain Vert’		Corvallis, USA	*Pyrus communis*	117;133
OHxF ^a^ 40		Corvallis, USA	*Pyrus communis*	115;122
OHxF ^a^ 112		Corvallis, USA	*Pyrus communis*	113;121
OHxF ^a^ 198		Corvallis, USA	*Pyrus communis*	113;121
OHXF ^a^ 230		Corvallis, USA	*Pyrus communis*	113;121
OHxF ^a^ 266		Corvallis, USA	*Pyrus communis*	115;118
OHxF ^a^ 267		Corvallis, USA	*Pyrus communis*	113;121
OHxF ^a^ 288		Corvallis, USA	*Pyrus communis*	113;121
OHXF ^a^ 333		Corvallis, USA	*Pyrus communis*	113;121
OHxF ^a^ 340		Corvallis, USA	*Pyrus communis*	115;123
OHxF ^a^ 361		Corvallis, USA	*Pyrus communis*	113;121
OHxF ^a^ 40		Corvallis, USA	*Pyrus communis*	113;121
OHXF ^a^ 51		Corvallis, USA	*Pyrus communis*	113;121
OHXF ^a^ 87		Corvallis, USA	*Pyrus communis*	113;121
OHxF ^a^ 9		Corvallis, USA	*Pyrus communis*	113;121
OHxF ^a^ 97		Corvallis, USA	*Pyrus communis*	113;121
‘Packham's Triumph’		PFR, NZ	*Pyrus communis*	113;-
‘Patrick Barry’		Corvallis, USA	*Pyrus communis*	**116**;-
‘Pyriam’		INRA, France	*Pyrus communis*	113;121
‘Pyrodwarf’		Corvallis, USA	*Pyrus communis*	114;**116**
QR 708-12		East Malling	*Pyrus communis*	**116**;-
QR 708-2		East Malling	*Pyrus communis*	115;-
QR 708-36		East Malling	*Pyrus communis*	113;**116**
‘Red Bartlett’		PFR, NZ	*Pyrus communis*	113;-
‘Red Pear’		Corvallis, USA	*Pyrus communis*	117;-
‘Rousselet de Reims’		Corvallis, USA	*Pyrus communis*	119;125
‘Sanguinole’		Corvallis, USA	*Pyrus communis*	113;128
‘Verbelu’		Corvallis, USA	*Pyrus communis*	112;117
‘Williams bon Chrétien’		PFR, NZ	*Pyrus communis*	111;113
OSU 3-6		INRA, France	*Pyrus betulifolia*	119;129
OPR 249		Corvallis, USA	*Pyrus calleryana*	109;124
OPR 255		INRA, France	*Pyrus calleryana*	125;-
OPR 264		Corvallis, USA	*Pyrus calleryana*	117;119
*Pyrus calleryana*		PFR, NZ	*Pyrus calleryana*	118;120
G28-120		INRA, France	*Pyrus nivalis*	113;124
G54-11		INRA, France	*Pyrus nivalis*	113;**116**
‘Poire Branche’		INRA, France	*Pyrus nivalis*	113;124
RV134		INRA, France	*Pyrus nivalis*	113;-
‘Naspati’		Corvallis, USA	*Pyrus pashia*	124;-
‘Kosui’		PFR, NZ	*Pyrus pyrifolia*	115;125
‘Sotoorihime’		Corvallis, USA	*Pyrus pyrifolia*	125;133
‘Eilon’		INRA, France	*Pyrus syriaca*	113;121
B II-3-25-27’		Corvallis, USA	*Pyrus ussuriensis*	**116**;129
‘Ping Ding Li’		Corvallis, USA	*Pyrus ussuriensis*	131;-
‘Tse Li’		Corvallis, USA	*Pyrus ussuriensis*	126;-
‘Lantai Jujuli’		Corvallis, USA	*Pyrus x sinkiangensis*	114;-

Pear rootstock cultivars are underlined. Hi01c04 is linked to *Dw1* in apple. The alleles are represented by the fragment size amplified and analysed using the ABI377 instrument. The 116 bp allele linked to low vigour in OHxLBJ is indicated in bold. Accessions with a OHxLBJ prefix are individuals from the OHxLBJ segregating population and are presented with their vigour and precocity phenotype. *PFR* The New Zealand Institute for Plant & Food Research Limited

^a^ Postman et al. [57] found that the ‘Old Home’ x ‘Farmingdale’ (OHxF) was actually a cross between ‘Old Home’ and ‘Bartlett’

This allele was also detected in 17 European pear cultivars (*P. communis* and *P. syriaca*), including *Pyrus* rootstocks such as ‘Pyriam’, ‘Fox’ and some rootstocks of the ‘Old Home’ x ‘Farmingdale’ (OHxF) series. The 116 bp fragment was more frequent in OHxLBJ individuals, conferring low vigour to the scion. This allele was also detected in the dwarfing rootstock ‘Pyrodwarf’, also derived from an OHxLBJ population.

## Discussion

Tree architecture and productivity are complex traits that are expressed only after several years of growth following grafting and are influenced by both genetics and environmental factors [44, 45]. Knowledge about genetic factors that confer rootstock-induced dwarfing of the scion in fruit trees is limited because of the difficulty in generating large segregating rootstock populations, as well as the requirement for robust and time-consuming phenotyping across multiple years, and the construction of dense genetic maps. Our systematic attention to these factors has enabled us to identify QTLs that control vigour and precocity in the grafted scion in pear rootstocks for the first time.

### TCA is a strong indicator of tree vigour

TCA was found to be a strong indicator for overall tree size in ‘Comice’ scions grafted to our seedling rootstocks, first becoming evident in year 2 and becoming stronger with each successive annual cycle, as previously reported for apple [46]. High correlations between the TCA and other vegetative growth traits, such as the height of the tree and the number of branches, substantiate our finding.

### QTLs controlling precocity and vigour

QTLs controlling both precocity and architectural traits were located on LG5 and LG6 of OH and LG6 for architectural traits segregating from LBJ. These QTLs are considered robust, as they were detected for successive years in the same position and often with the same marker having the highest LOD score. The co-location of all QTLs on LG5 and LG6, respectively, of OH indicates that the overall dwarfing effect is controlled by at least two loci. However, the variance explained by each QTL is low (4–18 %) indicating that the traits under investigation may be controlled by more loci than identified in this study. Furthermore, a strong environmental influence and a lack of replicates hampered the detection of major effect loci controlling precocity and architectural traits.

A small effect QTL for root suckering was detected on LG5 in the same genomic region as the architecture QTLs. Rootstocks of 69 % of the trees classified as dwarf produced suckers, indicating a strong correlation between dwarfing and suckering, and suggesting that the same physiological mechanisms might control both traits. We hypothesise that a reduced auxin transport from the scion to the roots, induced by *Pyrus* rootstocks, may promote root suckering and reduce scion vigour. This hypothesis is consistent with the findings that polar auxin transport in the xylem parenchyma inhibits suckering [47, 48], and that a reduced auxin transport in the rootstock stem occurs in dwarfed apple trees [6, 10].

### Synteny between apple and pear QTLs controlling scion vigour

The QTL controlling tree architecture and flowering on pear LG5 is in a genomic region orthologous to that of *Dw1*, which is the major locus for dwarfing conferred on apple scions by the ‘M9’ rootstock [12, 13]. These results are consistent with our hypothesis that orthologous loci in apple and pear control scion growth and precocity conferred by the rootstock. We found that the proximal marker flanking the *Dw1* locus in apple also segregates for dwarfing and precocity in pear, and that the 116 bp pear allele linked to low vigour in the rootstock mapping population is carried by the dwarfing pear rootstock ‘Pyrodwarf’. Our findings raise the possibility that the apple dwarfing locus *Dw1* and the OH LG5 QTL are derived from the same source, and therefore probably existed before the divergence of apple and pear. The conservation of synteny for QTLs involved in tree architecture has enabled us to align the genomic regions of interest. This will facilitate the identification of candidate genes for dwarfing in both species and enable the testing of our hypothesis of a common origin for this locus, either before the *Pyrus*-*Malus* speciation or due to hybridization.

Indeed, *Pyrus* and *Malus* are known to infrequently cross-hybridize [49, 50] and *P. communis* is sympatric with *M. sylvestris*, a related species that contributed genetically to the ancestry of modern *M.* x *domestica* [43, 51–53], including the dwarfing rootstock ‘M9’. It is interesting to note that a second pear QTL corresponding to apple *Dw2* was not detected on LG11 in the present study. Phenotypic analysis of scions grafted to segregating rootstock populations has demonstrated that the combination of *Dw1* and *Dw2* confers the greatest degree of rootstock-induced dwarfing in apple rootstocks [12, 14].

### Tree size and precocity

We found that precocious and non-precocious trees differed significantly in the number of sylleptic branches grown in the first year, with a lower sylleptic branch number correlating with a delay in flowering. Although studies in pear indicate that the scion cultivar has a greater influence on sylleptic shoot formation than the rootstock [54, 55], Watson et al. [56] found that both the rootstock and the scion influence the number of first-year sylleptic branches. In this study we have confirmed the influence of the rootstock on early branch development in pear; however, we could not evaluate the effect of the scion. Watson et al. [56] also found that increased flowering did not result in early growth reduction in pear, and they suggested that the difference between the apple and pear rootstock dwarfing effects might lie in the length of the juvenile period.

The QTL conferring reduced sylleptic branching, tree size and TCA in pear co-locates with the QTL on LG5 conferring precocity. However, the effects of the detected QTLs are in trans, unlike the situation in apple, meaning that a smaller tree takes longer to flower. In terms of breeding, this means that it is difficult to breed for rootstocks conferring both reduced tree size and precocity to the scion cultivar. However, 14 % of the trees in our segregating rootstock population did exhibit low vigour and precocity in their grafted scions.

Assuming the application of marker assisted selection (MAS) on the basis of the ss475878191 AA allelotype, only 11 % of the individuals would have the desired low vigour and precocious phenotype. However, selection for the AB allelotype would result in a slightly higher percentage (16 %) of individuals with both desired traits in the progeny. Hence, selecting for the ss475878191 AB allele would increase the proportion of rootstocks in a breeding population that confer both precocity and a reduced vigour to the scion. However, the percentage of rootstocks conferring low vigour and precocity was generally low (14 %). It is noteworthy that in this respect, our findings for the pear rootstock QTLs differ from the effects of the apple QTL on LG5 which confers both precocity and a reduced tree size. The dwarfing effect of ‘M9’ apple rootstocks is correlated with an increase in the proportion of floral buds relative to vegetative buds and sylleptic shoots that develop within the first year of growth [11, 15]. The up-regulation of key flowering genes in ‘M9’ rootstocks may be responsible for this shift from vegetative to floral development. As a high proportion of axillary floral shoots leads to reduced vegetative growth in the next growth cycle, the tree becomes more dwarfed over time. A substantial difference between flowering in apple scions grafted onto ‘M9’ and the pear scions in this study was the position of the floral buds on the developing tree. In apple, flowering occurs in axillary and terminal buds, and, as the tree ages, is more common on 2-year-old spurs. In pear, flowering mostly occurs on two-year-old spurs and terminal buds. This biological difference has profound implications to the subsequent development of tree architecture. Apple scions grafted onto ‘M9’ flower at the beginning of the second year of growth after grafting, while pear scions on pear rootstocks do not flower until the third year of growth and thus have a longer period of vegetative growth before flowering. In pear, an increase in branching in years 1 and 2 would generate the potential for more spurs for flowering in year 3, and hence the relationship between early vegetative vigour and flowering would be opposite to that observed in apple. No QTL was detected for the percentage of axillary inflorescence in this study. It might be predicted that crossing a rootstock that increases the number of axillary buds on the scion cultivar with OH might result in a dwarfing pear rootstock, similar to ‘M9’, conferring both low vigour and precocity to the scion.

## Conclusion

In this study we detected the first pear rootstock QTLs associated with control of architectural and flowering traits in scions. Furthermore, we found that these orthologous loci control scion growth and precocity in apple and pear rootstocks. These findings will facilitate the identification of candidate genes for control of scion traits by rootstocks in both species. Future research may focus on finding the common origin for the dwarfing locus in apple and pear. The application of our results in pear rootstock breeding may assist in developing a marker for MAS selection for breeding a pear rootstock that confers both vigour control and precocity to the grafted scion cultivar.

### Availability of supporting data

Genetic map data used in this study can be found in Montanari et al. [33]. Phenotypic data is available on request.

## Additional files

Additional file 1: Table S1. Pearson correlation (first cell) and P-value (second cell) of all the traits measured over four years in the ‘Old Home’ x ‘Louise Bonne de Jersey’ OHxLBJ segregating pear population. Branches: branches per tree; Height: total tree height; Inflorescence: inflorescences per tree; Nodes: nodes per tree; Spurs: spurs per tree; TCAtrunk: trunk cross-sectional area 20 cm above graft unit; TCAroot: TCA of rootstock; TCAsec: TCA secondary growth of the main axis; TCAtert: TCA tertiary growth of the main axis. (PDF 164 kb) Additional file 2: Figure S1. Alignment of linkage groups from ‘Louise Bonne de Jersey’ (LBJ) and ‘Old Home’ (OH) pears with the maps of ‘Moonglow’ (Moon) and PEAR1 (Montanari et al., 2013). The markers are named using the NCBI dbSNP accessions and their positions are indicated in centiMorgan. Microsatellite markers mapped in the ‘Moonglow’ x PEAR1 population are underlined. The linkage group (LG) numbering system is consistent with the apple LG numbering. Identified QTLs are shown with blue symbols coming from OH and brown symbols from LBJ. The *Dw1* flanking marker Hi01c04 (underlined and red) mapped to LG5 of OH. (PDF 306 kb)

## Abbreviations

ANOVA: Analysis of variance
bp: Base pair
cM: CentiMorgan
CTAB: Cetyltrimethyl ammonium bromide
DNA: Deoxyribonucleic acid
*Dw1*: *Dwarfing locus 1*
*Dw2*: *Dwarfing locus 2*
*FT*: *FLOWERING LOCUS*
IM: Interval mapping
INRA: Institut National de Recherche Agronomique
IRSC: International RosBREED SNP Consortium
kbp: Kilo base pairs
LBJ: ‘Louise Bonne de Jersey’
LG: Linkage group
LNG: Length of the new main axis growth
LOD: Logarithm of odds
M9: ‘Malling 9’
MAS: Marker assisted selection
Mbp: Mega base pairs
MQM: Multiple QTL mapping
NCBI: National Centre for Biotechnology Information
OH: ‘Old Home’
OHxF: ‘Old Home’ x ‘Farmingdale’
PCR: Polymerase chain reaction
PFR: Plant and Food Research
QC: ‘Quince C’
QTL: Quantitative trait locus
SNP: Single nucleotide polymorphism
SSR: Simple sequence repeat
TCA: Trunk cross-sectional area
