# Supplementary material for: Genetic control of pear rootstock-induced dwarfing and precocity is linked to a chromosomal region syntenic to the apple Dw1 loci
Source: BMC Plant Biol. 2015 Sep 22;15:230. doi: 10.1186/s12870-015-0620-4 (PMC4580296; doi:10.1186/s12870-015-0620-4)
Supplement: Additional file 1: Table S1. — Pearson correlation (first cell) and P-value (second cell) of all the traits measured over four years in the ‘Old Home’ x ‘Louise Bonne de Jersey’ OHxLBJ segregating pear population. Branches: branches per tree; Height: total tree height; Inflorescence: inflorescences per tree; Nodes: nodes per tree; Spurs: spurs per tree; TCAtrunk: trunk cross-sectional area 20 cm above graft unit; TCAroot: TCA of rootstock; TCAsec: TCA secondary growth of the main axis; TCAtert: TCA tertiary growth of the main axis. (PDF 164 kb) [file 12870_2015_620_MOESM1_ESM.pdf]

Additional file 1: Table S1: Pearson correlation (first cell) and P-value (second cell) of all the traits measured over four years in the 'Old Home' x 'Louise Bonne de Jersey' OHxLBJ segregating pear population. Branches: branches per tree; height: total tree height; inflorescence: inflorescences per tree; nodes: nodes per tree; Spurs: spurs per tree; TCAtrunk: trunk cross-sectional area 20 cm above graft unit; TCAroot: TCA of rootstock; TCAsec: TCA secondary growth of the main axis; TCAtert: TCA tertiary growth of the main axis.

	Branches year 1	Branches year 2	Branches year 3	Height year 1	Height year 2	Height year 3	Inflor-escence year 3	Inflor-escence year 4	LNG year 2	LNG year 3	LNG year 4	Nodes year 1	Nodes year 2	Spurs year 1	Spurs year 2	Spurs year 3	TCA year 1	TCA year 2	TCA year 3	TCA year 4	TCA root year 3	TCA root year 4	TCA sec year 3
Branches	0.757																						
year 2	0.000																						
Branches	0.599	0.635																					
year 3	0.000	0.000																					
Height	0.371	0.534	0.364																				
year 1	0.000	0.000	0.000																				
Height	0.552	0.795	0.524	0.898																			
year2	0.000	0.000	0.000	0.000																			
Height	0.485	0.467	0.614	0.529	0.569																		
year 3	0.000	0.000	0.000	0.000	0.000																		
Inflorescence	0.366	0.456	0.144	0.296	0.407	0.192																	
year 3	0.000	0.000	0.017	0.000	0.000	0.002																	
Inflorescence	0.318	0.398	0.360	0.342	0.389	0.302	0.546																
year 4	0.000	0.000	0.000	0.000	0.000	0.000	0.000																
LNG	0.263	0.085	0.276	-0.106	-0.021	0.559	-0.064	0.061															
year 2	0.000	0.174	0.000	0.089	0.740	0.000	0.295	0.326															
LNG	0.336	0.301	0.513	0.121	0.224	0.761	0.134	0.162	0.251														
year 3	0.000	0.000	0.000	0.050	0.000	0.000	0.025	0.007	0.000														
LNG	0.274	0.244	0.406	0.074	0.117	0.443	0.074	0.113	0.162	0.554													
year 4	0.000	0.000	0.000	0.233	0.060	0.000	0.218	0.061	0.008	0.000													
Nodes	0.327	0.424	0.285	0.800	0.687	0.348	0.211	0.307	-0.137	0.069	0.048												
year 1	0.000	0.000	0.000	0.000	0.000	0.000	0.001	0.000	0.027	0.267	0.441												
Nodes	0.165	-0.033	0.134	-0.210	-0.175	0.381	-0.157	-0.010	0.818	0.179	0.217	-0.235											
year 2	0.007	0.600	0.031	0.001	0.005	0.000	0.010	0.876	0.000	0.003	0.000	0.000											
Spurs	0.189	0.150	0.158	0.295	0.238	0.146	0.107	0.194	-0.121	0.101	0.054	0.418	-0.213										
year 1	0.002	0.016	0.012	0.000	0.000	0.020	0.083	0.002	0.051	0.103	0.390	0.000	0.001										
Spurs	0.797	0.739	0.563	0.322	0.529	0.463	0.379	0.310	0.269	0.325	0.289	0.267	0.179	0.031									
year 2	0.000	0.000	0.000	0.000	0.000	0.000	0.000	0.000	0.000	0.000	0.000	0.000	0.003	0.615									
Spurs	0.721	0.815	0.646	0.486	0.659	0.562	0.430	0.553	0.181	0.387	0.337	0.448	0.061	0.191	0.732								
year 3	0.000	0.000	0.000	0.000	0.000	0.000	0.000	0.000	0.003	0.000	0.000	0.000	0.327	0.002	0.000								
TCAtrunk	0.740	0.720	0.576	0.433	0.580	0.470	0.358	0.310	0.183	0.305	0.195	0.363	0.076	0.134	0.638	0.719							
year 1	0.000	0.000	0.000	0.000	0.000	0.000	0.000	0.000	0.003	0.000	0.002	0.000	0.225	0.031	0.000	0.000							
TCAtrunk	0.606	0.629	0.611	0.295	0.422	0.427	0.302	0.370	0.158	0.353	0.323	0.263	0.258	0.031	0.608	0.663	0.665						
year 2	0.000	0.000	0.000	0.000	0.000	0.000	0.000	0.000	0.010	0.000	0.000	0.000	0.000	0.617	0.000	0.000	0.000						
TCAtrunk	0.698	0.733	0.834	0.409	0.558	0.714	0.320	0.414	0.314	0.620	0.462	0.331	0.186	0.129	0.701	0.764	0.733	0.752					
year 3	0.000	0.000	0.000	0.000	0.000	0.000	0.000	0.000	0.000	0.000	0.000	0.000	0.002	0.037	0.000	0.000	0.000	0.000					
TCAtrunk	0.666	0.675	0.812	0.352	0.496	0.724	0.281	0.344	0.341	0.653	0.526	0.261	0.226	0.101	0.633	0.701	0.662	0.698	0.964				
year 4	0.000	0.000	0.000	0.000	0.000	0.000	0.000	0.000	0.000	0.000	0.000	0.000	0.000	0.104	0.000	0.000	0.000	0.000	0.000				
TCAroot	0.697	0.735	0.795	0.430	0.574	0.680	0.316	0.436	0.276	0.569	0.446	0.348	0.168	0.163	0.701	0.775	0.674	0.714	0.921	0.889			
year 3	0.000	0.000	0.000	0.000	0.000	0.000	0.000	0.000	0.000	0.000	0.000	0.000	0.006	0.008	0.000	0.000	0.000	0.000	0.000	0.000			
TCAroot	0.653	0.694	0.788	0.377	0.515	0.685	0.269	0.364	0.292	0.601	0.503	0.284	0.191	0.135	0.638	0.718	0.625	0.681	0.919	0.931	0.958		
year 4	0.000	0.000	0.000	0.000	0.000	0.000	0.000	0.000	0.000	0.000	0.000	0.000	0.002	0.030	0.000	0.000	0.000	0.000	0.000	0.000	0.000		
TCAsec	0.443	0.396	0.618	0.139	0.254	0.704	0.143	0.267	0.600	0.609	0.436	0.114	0.486	0.014	0.426	0.492	0.395	0.535	0.693	0.725	0.638	0.684	
year 3	0.000	0.000	0.000	0.025	0.000	0.000	0.017	0.000	0.000	0.000	0.000	0.065	0.000	0.823	0.000	0.000	0.000	0.000	0.000	0.000	0.000	0.000	
TCAtert	0.422	0.380	0.567	0.140	0.261	0.758	0.198	0.254	0.464	0.795	0.458	0.105	0.395	0.018	0.413	0.458	0.395	0.437	0.652	0.679	0.604	0.633	0.791
year 3	0.000	0.000	0.000	0.024	0.000	0.000	0.001	0.000	0.000	0.000	0.000	0.091	0.000	0.770	0.000	0.000	0.000	0.000	0.000	0.000	0.000	0.000	0.000
