# Supplementary material for: Genetic control of pear rootstock-induced dwarfing and precocity is linked to a chromosomal region syntenic to the apple Dw1 loci
Source: BMC Plant Biol. 2015 Sep 22;15:230. doi: 10.1186/s12870-015-0620-4 (PMC4580296; doi:10.1186/s12870-015-0620-4)
Supplement: Additional file 2: Figure S1. — Alignment of linkage groups from ‘Louise Bonne de Jersey’ (LBJ) and ‘Old Home’ (OH) pears with the maps of ‘Moonglow’ (Moon) and PEAR1 (Montanari et al., 2013). The markers are named using the NCBI dbSNP accessions and their positions are indicated in centiMorgan. Microsatellite markers mapped in the ‘Moonglow’ x PEAR1 population are underlined. The linkage group (LG) numbering system is consistent with the apple LG numbering. Identified QTLs are shown with blue symbols coming from OH and brown symbols from LBJ. The Dw1 flanking marker Hi01c04 (underlined and red) mapped to LG5 of OH. (PDF 306 kb) [file 12870_2015_620_MOESM2_ESM.pdf]

Additional file 2: Figure S1: Alignment of linkage groups from 'Louise Bonne de Jersey' (LBJ) and 'Old Home' (OH) pears with the maps of 'Moonglow' (Moon) and PEAR1 (Montanari *et al.*, 2013). The markers are named using the NCBI dbSNP accessions and their positions are indicated in centiMorgan. Microsatellite markers mapped in the 'Moonglow' x PEAR1 population are underlined. The linkage group (LG) numbering system is consistent with the apple LG numbering. Identified QTLs are shown with blue symbols coming from OH and brown symbols from LBJ. The *Dw1* flanking marker Hi01c04 (underlined and red) mapped to LG5 of OH.

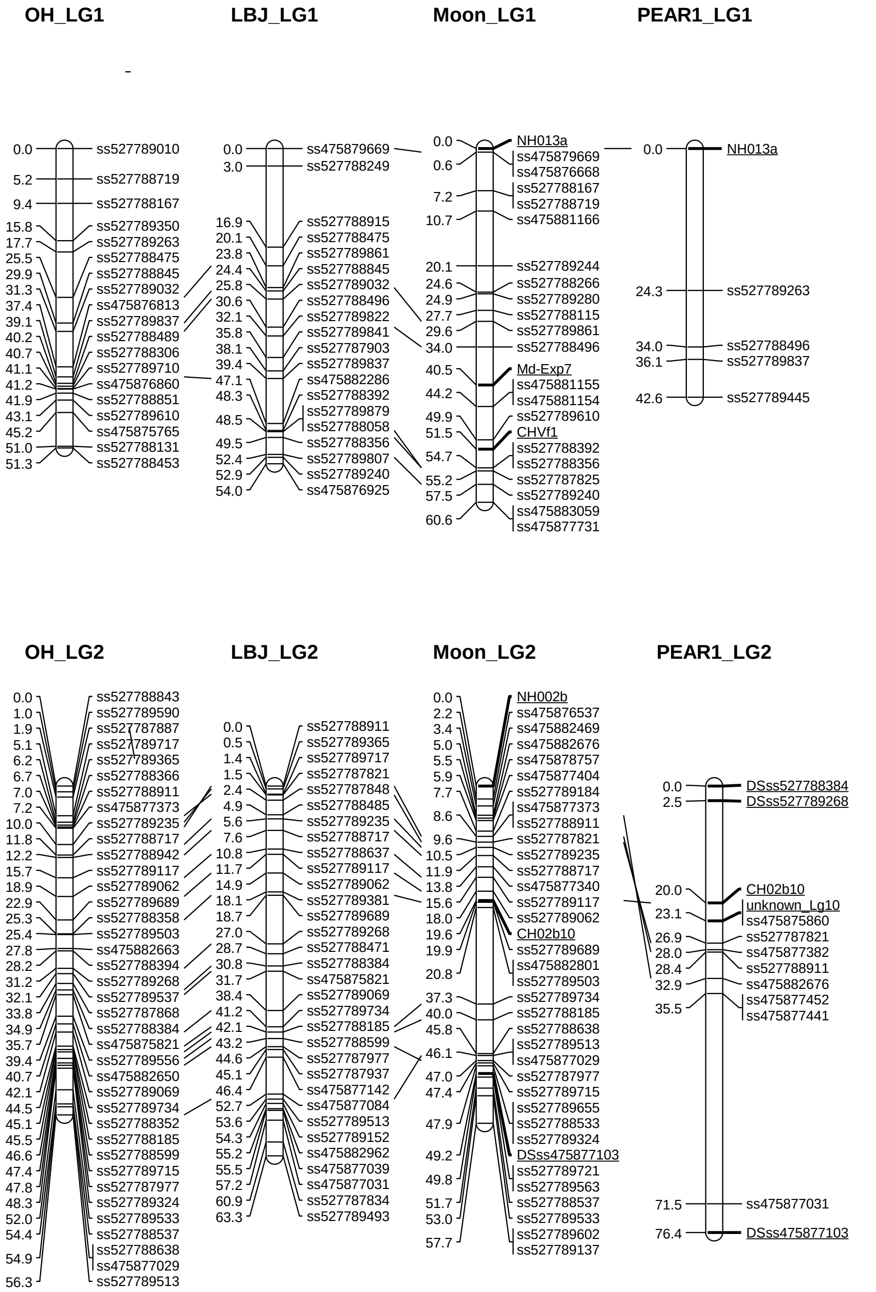

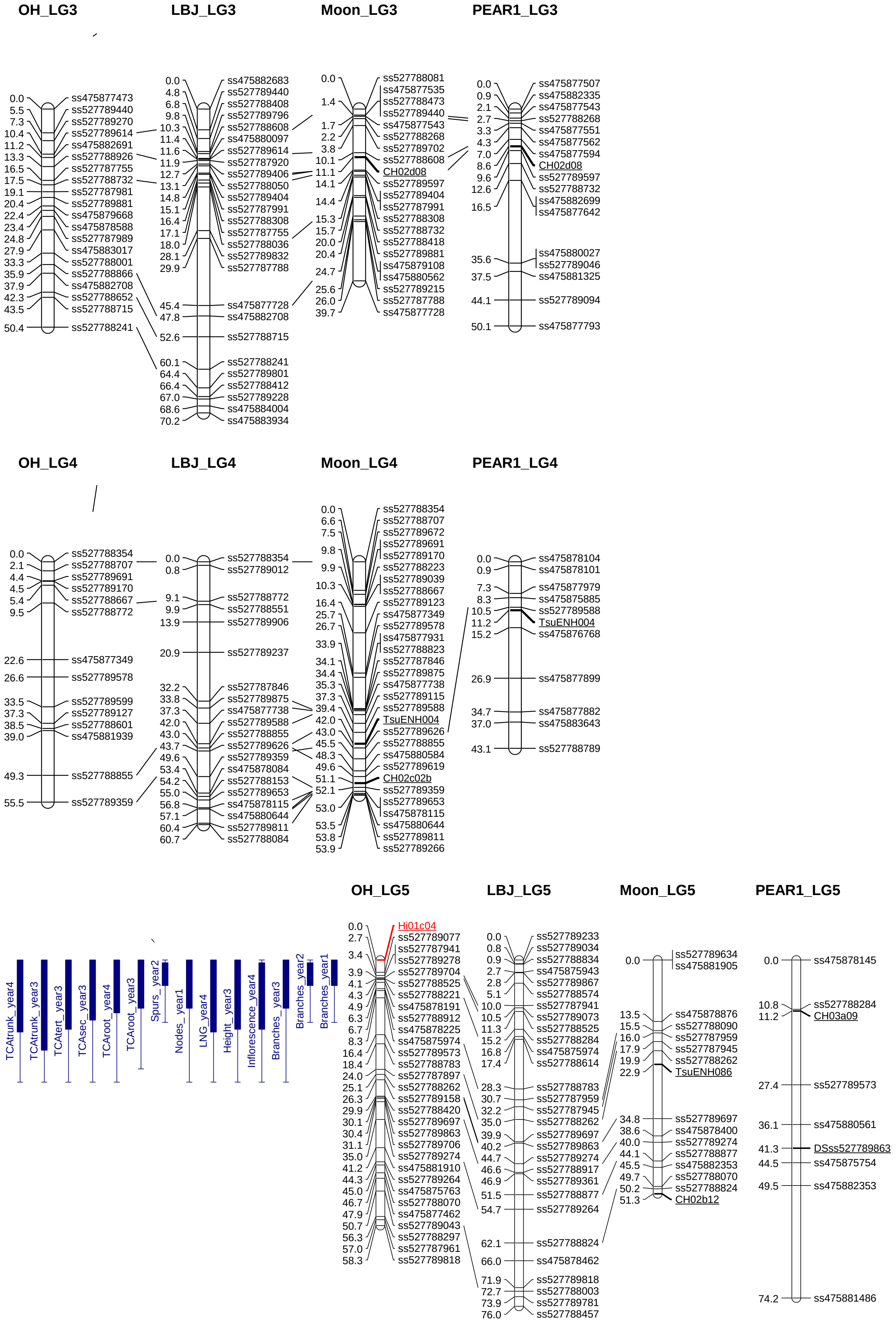
OH_LG3
0.0 ss475877473
5.5 ss527789440
7.3 ss527789270
10.4 ss527789614
11.2 ss475882691
13.3 ss527788926
16.5 ss527787755
17.5 ss527788732
19.1 ss527787981
20.4 ss527789881
22.4 ss475879668
23.4 ss475878588
24.8 ss527787989
27.9 ss475883017
33.3 ss527788001
35.9 ss527788866
37.9 ss475882708
42.3 ss527788652
43.5 ss527788715
50.4 ss527788241
LBJ_LG3
0.0 ss475882683
4.8 ss527789440
6.8 ss527788408
9.8 ss527789796
10.3 ss527788608
11.4 ss475880097
11.6 ss527789614
11.9 ss527787920
12.7 ss527789406
13.1 ss527788050
14.8 ss527789404
15.1 ss527787991
16.4 ss527788308
17.1 ss527787755
18.0 ss527788036
28.1 ss527789832
29.9 ss527787788
45.4 ss475877728
47.8 ss475882708
52.6 ss527788715
60.1 ss527788241
64.4 ss527789801
66.4 ss527788412
67.0 ss527789228
68.6 ss475884004
70.2 ss475883934
Moon_LG3
0.0 ss527788081
ss475877535
1.4 ss527788473
ss527789440
1.7 ss475877543
2.2 ss527788268
3.8 ss527789702
10.1 ss527788608
11.1 CH02d08
14.1 ss527789597
ss527789404
14.4 ss527787991
ss527788308
15.3 ss527788732
15.7 ss527788418
20.0 ss527789881
20.4 ss475879108
24.7 ss475880562
25.6 ss527789215
26.0 ss527787788
39.7 ss475877728
PEAR1_LG3
0.0 ss475877507
0.9 ss475882335
2.1 ss475877543
2.7 ss527788268
3.3 ss475877551
4.3 ss475877562
7.0 ss475877594
8.6 CH02d08
9.6 ss527789597
12.6 ss527788732
16.5 ss475882699
ss475877642
35.6 ss475880027
ss527789046
37.5 ss475881325
44.1 ss527789094
50.1 ss475877793
OH_LG4
0.0 ss527788354
2.1 ss527788707
4.4 ss527789691
4.5 ss527789170
5.4 ss527788667
9.5 ss527788772
22.6 ss475877349
26.6 ss527789578
33.5 ss527789599
37.3 ss527789127
38.5 ss527788601
39.0 ss475881939
49.3 ss527788855
55.5 ss527789359
LBJ_LG4
0.0 ss527788354
0.8 ss527789012
9.1 ss527788772
9.9 ss527788551
13.9 ss527789906
20.9 ss527789237
32.2 ss527787846
33.8 ss527789875
37.3 ss475877738
42.0 ss527789588
43.0 ss527788855
43.7 ss527789626
49.6 ss527789359
53.4 ss475878084
54.2 ss527788153
55.0 ss527789653
56.8 ss475878115
57.1 ss475880644
60.4 ss527789811
60.7 ss527788084
Moon_LG4
0.0 ss527788354
6.6 ss527788707
7.5 ss527789672
9.8 ss527789691
9.9 ss527789170
10.3 ss527788223
16.4 ss527789039
25.7 ss527788667
26.7 ss527789123
33.9 ss475877349
34.1 ss527789578
34.4 ss475877931
35.3 ss527788823
37.3 ss527787846
39.4 ss527789875
42.0 ss475877738
43.0 ss527789115
45.5 ss527789588
48.3 TsuENH004
49.6 ss527789626
51.1 ss527788855
52.1 ss475880584
53.0 ss527789619
53.5 CH02c02b
53.8 ss527789359
53.9 ss527789653
ss475878115
ss475880644
ss527789811
ss527789266
PEAR1_LG4
0.0 ss475878104
0.9 ss475878101
7.3 ss475877979
8.3 ss475875885
10.5 ss527789588
11.2 TsuENH004
15.2 ss475876768
26.9 ss475877899
34.7 ss475877882
37.0 ss475883643
43.1 ss527788789
TCAtrunk_year4
TCAtrunk_year3
TCAtert_year3
TCAsec_year3
TCAroot_year4
TCAroot_year3
Spurs_year2
Nodes_year1
LNG_year4
Height_year3
Inflorescence_year4
Branches_year3
Branches_year2
Branches_year1
OH_LG5
0.0 Hi01c04
2.7 ss527789077
3.4 ss527787941
3.9 ss527789278
4.1 ss527789704
4.3 ss527788525
4.9 ss527788221
6.3 ss475878191
6.7 ss527788912
8.3 ss475878225
16.4 ss475875974
18.4 ss527789573
24.0 ss527788783
25.1 ss527787897
26.3 ss527788262
29.9 ss527789158
30.1 ss527788420
30.4 ss527789697
31.1 ss527789863
35.0 ss527789706
41.2 ss527789274
44.3 ss475881910
45.0 ss527789264
46.7 ss475875763
47.9 ss527788070
50.7 ss475877462
56.3 ss527789043
57.0 ss527788297
58.3 ss527787961
ss527789818
LBJ_LG5
0.0 ss527789233
0.8 ss527789034
0.9 ss527788834
2.7 ss475875943
2.8 ss527789867
5.1 ss527788574
10.0 ss527787941
10.5 ss527789073
11.3 ss527788525
15.2 ss527788284
16.8 ss475875974
17.4 ss527788614
28.3 ss527788783
30.7 ss527787959
32.2 ss527787945
35.0 ss527788262
39.9 ss527789697
40.2 ss527789863
44.7 ss527789274
46.6 ss527788917
46.9 ss527789361
51.5 ss527788877
54.7 ss527789264
62.1 ss527788824
66.0 ss475878462
71.9 ss527789818
72.7 ss527788003
73.9 ss527789781
76.0 ss527788457
Moon_LG5
0.0 ss527789634
ss475881905
13.5 ss475878876
15.5 ss527788090
16.0 ss527787959
17.9 ss527787945
19.9 ss527788262
22.9 TsuENH086
34.8 ss527789697
38.6 ss475878400
40.0 ss527789274
44.1 ss527788877
45.5 ss475882353
49.7 ss527788070
50.2 ss527788824
51.3 CH02b12
PEAR1_LG5
0.0 ss475878145
10.8 ss527788284
11.2 CH03a09
27.4 ss527789573
36.1 ss475880561
41.3 DSss527789863
44.5 ss475875754
49.5 ss475882353
74.2 ss475881486

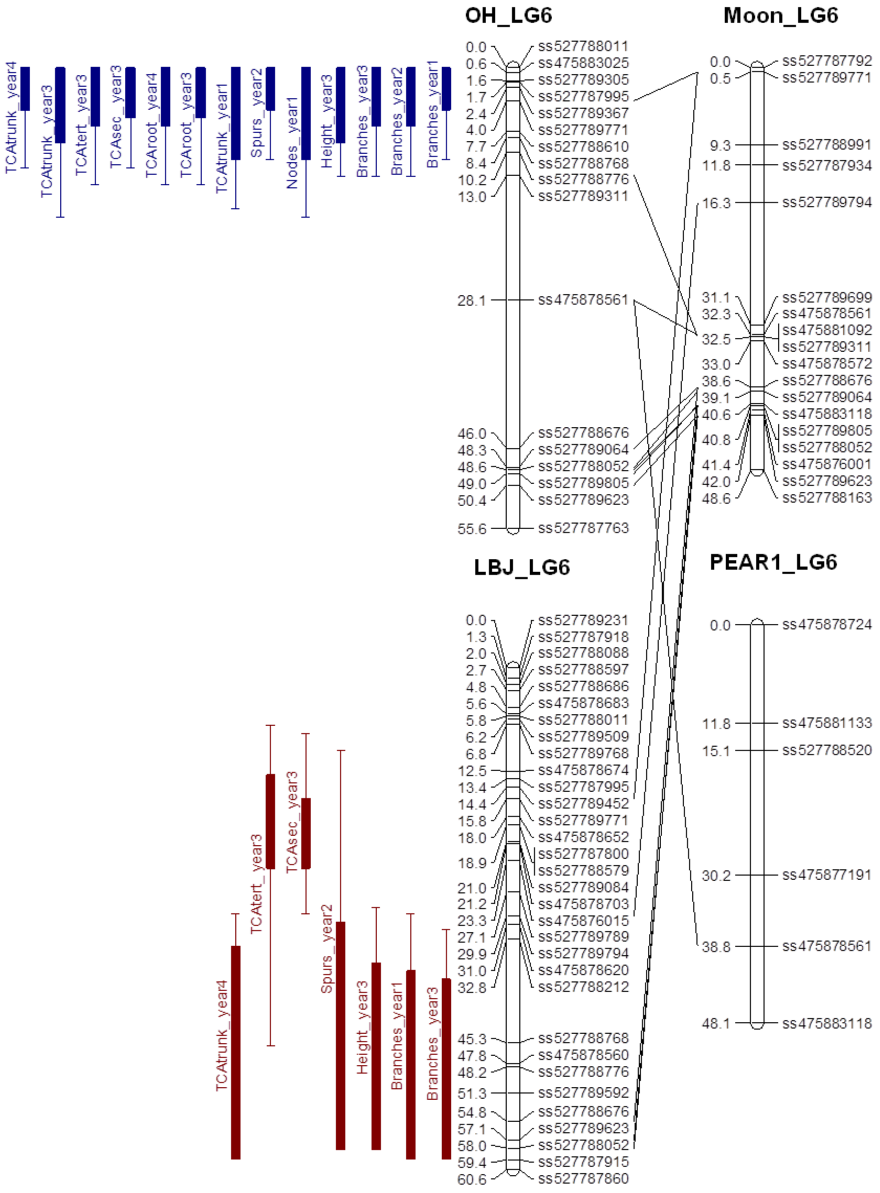
OH_LG6
Moon_LG6
LBJ_LG6
PEAR1_LG6
TCAtrunk_year4
TCAtrunk_year3
TCAtert_year3
TCAsec_year3
TCAroot_year4
TCAroot_year3
TCAtrunk_year1
Spurs_year2
Nodes_year1
Height_year3
Branches_year3
Branches_year2
Branches_year1
Spurs_year2
Height_year3

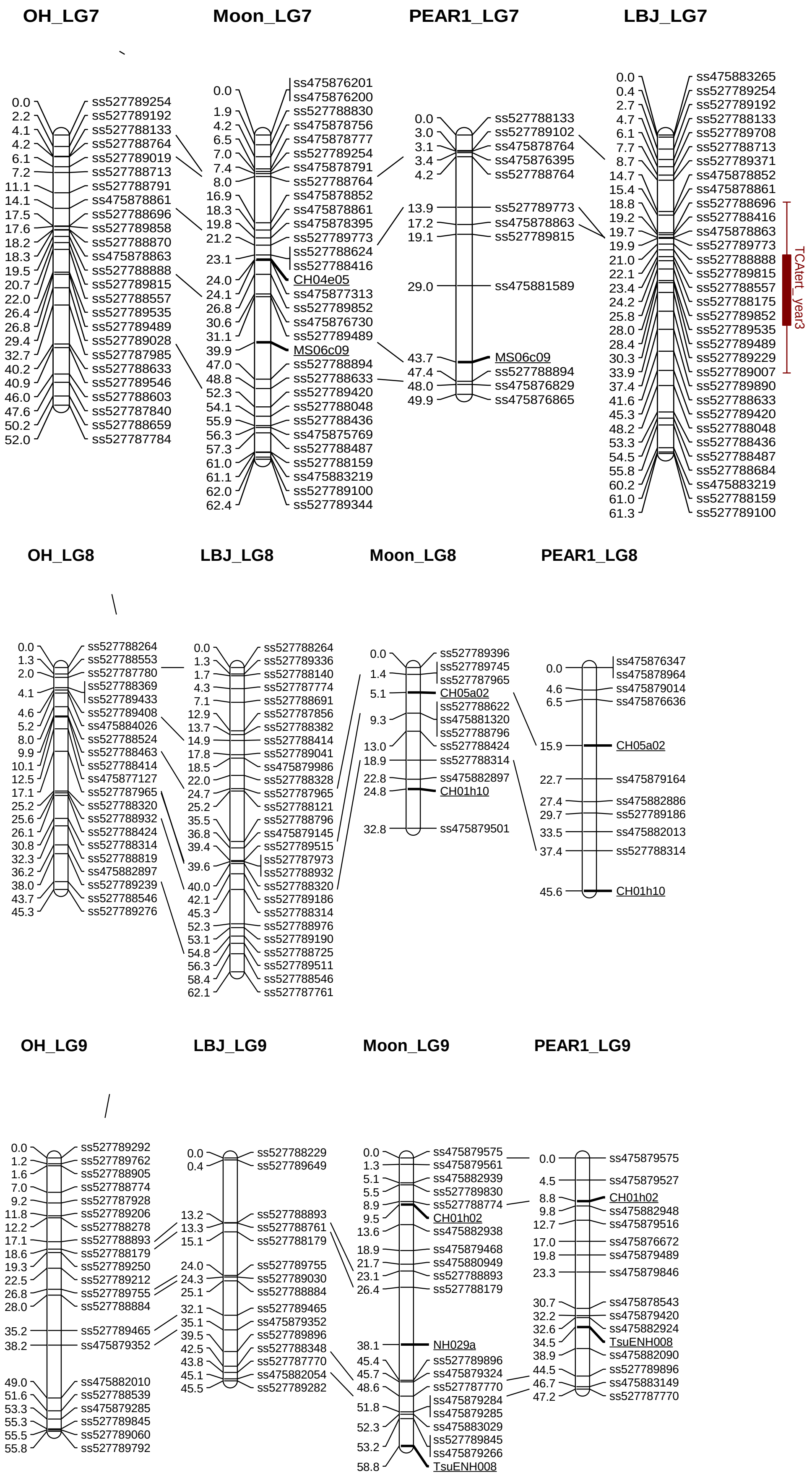

OH_LG7
Moon_LG7
PEAR1_LG7
LBJ_LG7
TCAtert_year3
OH_LG8
LBJ_LG8
Moon_LG8
PEAR1_LG8
OH_LG9
LBJ_LG9
Moon_LG9
PEAR1_LG9

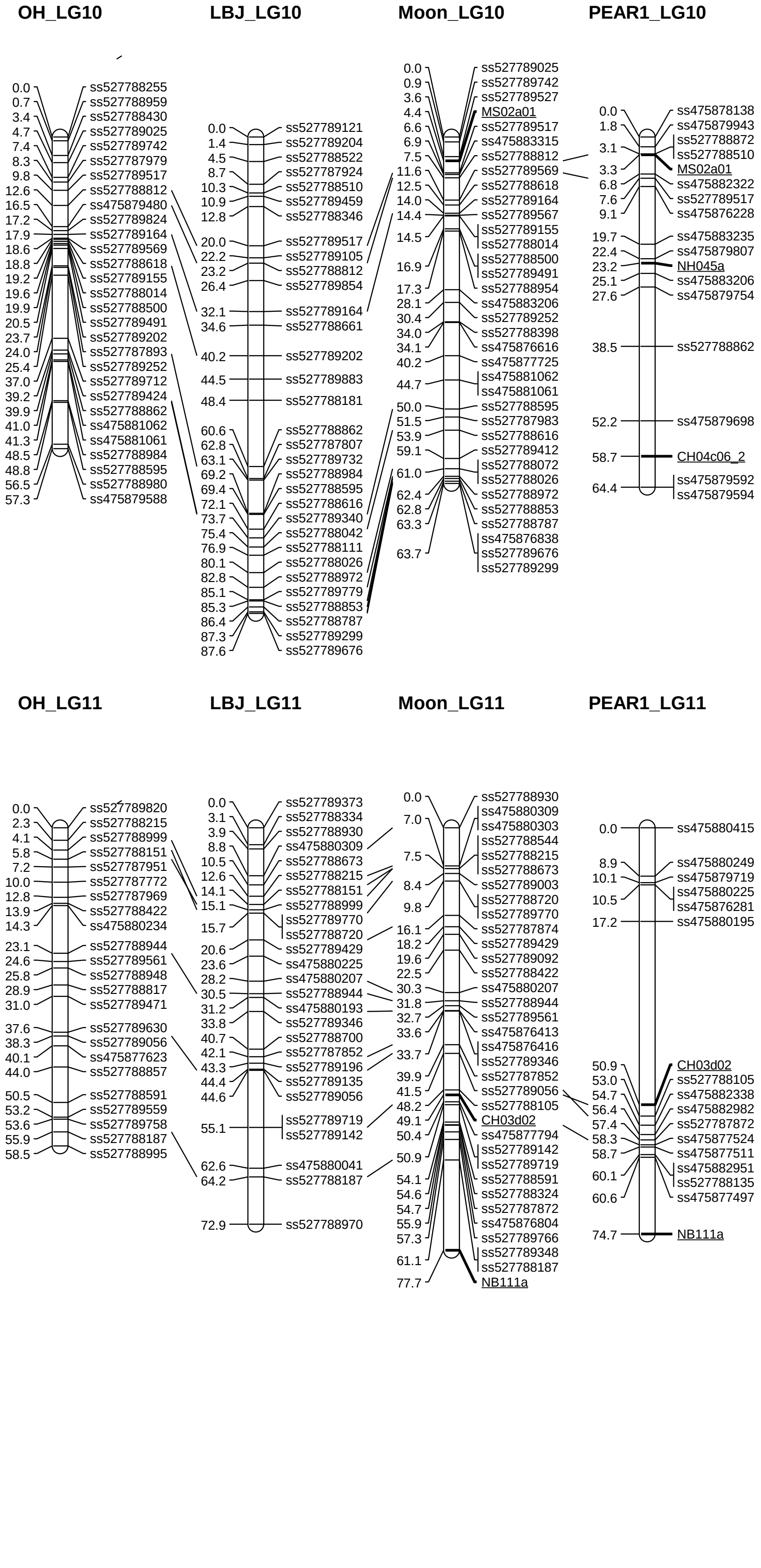
OH_LG10
0.0 ss527788255
0.7 ss527788959
3.4 ss527788430
4.7 ss527789025
7.4 ss527789742
8.3 ss527787979
9.8 ss527789517
12.6 ss527788812
16.5 ss475879480
17.2 ss527789824
17.9 ss527789164
18.6 ss527789569
18.8 ss527788618
19.2 ss527789155
19.6 ss527788014
19.9 ss527788500
20.5 ss527789491
23.7 ss527789202
24.0 ss527787893
25.4 ss527789252
37.0 ss527789712
39.2 ss527789424
39.9 ss527788862
41.0 ss475881062
41.3 ss475881061
48.5 ss527788984
48.8 ss527788595
56.5 ss527788980
57.3 ss475879588
LBJ_LG10
0.0 ss527789121
1.4 ss527789204
4.5 ss527788522
8.7 ss527787924
10.3 ss527788510
10.9 ss527789459
12.8 ss527788346
20.0 ss527789517
22.2 ss527789105
23.2 ss527788812
26.4 ss527789854
32.1 ss527789164
34.6 ss527788661
40.2 ss527789202
44.5 ss527789883
48.4 ss527788181
60.6 ss527788862
62.8 ss527787807
63.1 ss527789732
69.2 ss527788984
69.4 ss527788595
72.1 ss527788616
73.7 ss527789340
75.4 ss527788042
76.9 ss527788111
80.1 ss527788026
82.8 ss527788972
85.1 ss527789779
85.3 ss527788853
86.4 ss527788787
87.3 ss527789299
87.6 ss527789676
Moon_LG10
0.0 0.9 3.6 4.4 6.6 6.9 7.5 11.6 12.5 14.0 14.4 14.5 16.9 17.3 28.1 30.4 34.0 34.1 40.2 44.7 50.0 51.5 53.9 59.1 61.0 62.4 62.8 63.3 63.7
ss527789025 ss527789742 ss527789527 MS02a01 ss527789517 ss475883315 ss527788812 ss527789569 ss527788618 ss527789164 ss527789567 ss527789155 ss527788014 ss527788500 ss527789491 ss527788954 ss475883206 ss527789252 ss527788398 ss475876616 ss475877725 ss475881062 ss475881061 ss527788595 ss527787983 ss527788616 ss527789412 ss527788072 ss527788026 ss527788972 ss527788853 ss527788787 ss475876838 ss527789676 ss527789299
PEAR1_LG10
0.0 1.8 3.1 3.3 6.8 7.6 9.1 19.7 22.4 23.2 25.1 27.6 38.5 52.2 58.7 64.4
ss475878138 ss475879943 ss527788872 ss527788510 MS02a01 ss475882322 ss527789517 ss475876228 ss475883235 ss475879807 NH045a ss475883206 ss475879754 ss527788862 ss475879698 CH04c06_2 ss475879592 ss475879594
OH_LG11
0.0 ss527789820
2.3 ss527788215
4.1 ss527788999
5.8 ss527788151
7.2 ss527787951
10.0 ss527787772
12.8 ss527787969
13.9 ss527788422
14.3 ss475880234
23.1 ss527788944
24.6 ss527789561
25.8 ss527788948
28.9 ss527788817
31.0 ss527789471
37.6 ss527789630
38.3 ss527789056
40.1 ss475877623
44.0 ss527788857
50.5 ss527788591
53.2 ss527789559
53.6 ss527789758
55.9 ss527788187
58.5 ss527788995
LBJ_LG11
0.0 3.1 3.9 8.8 10.5 12.6 14.1 15.1 15.7 20.6 23.6 28.2 30.5 31.2 33.8 40.7 42.1 43.3 44.4 44.6 55.1 62.6 64.2 72.9
ss527789373 ss527788334 ss527788930 ss475880309 ss527788673 ss527788215 ss527788151 ss527788999 ss527789770 ss527788720 ss527789429 ss475880225 ss475880207 ss527788944 ss475880193 ss527789346 ss527788700 ss527787852 ss527789196 ss527789135 ss527789056 ss527789719 ss527789142 ss475880041 ss527788187 ss527788970
Moon_LG11
0.0 7.0 7.5 8.4 9.8 16.1 18.2 19.6 22.5 30.3 31.8 32.7 33.6 33.7 39.9 41.5 48.2 49.1 50.4 50.9 54.1 54.6 54.7 55.9 57.3 61.1 77.7
ss527788930 ss475880309 ss475880303 ss527788544 ss527788215 ss527788673 ss527789003 ss527788720 ss527789770 ss527787874 ss527789429 ss527789092 ss527788422 ss475880207 ss527788944 ss527789561 ss475876413 ss475876416 ss527789346 ss527787852 ss527789056 ss527788105 CH03d02 ss475877794 ss527789142 ss527789719 ss527788591 ss527788324 ss527787872 ss475876804 ss527789766 ss527789348 ss527788187 NB111a
PEAR1_LG11
0.0 8.9 10.1 10.5 17.2 50.9 53.0 54.7 56.4 57.4 58.3 58.7 60.1 60.6 74.7
ss475880415 ss475880249 ss475879719 ss475880225 ss475876281 ss475880195 CH03d02 ss527788105 ss475882338 ss475882982 ss527787872 ss475877524 ss475877511 ss475882951 ss527788135 ss475877497 NB111a

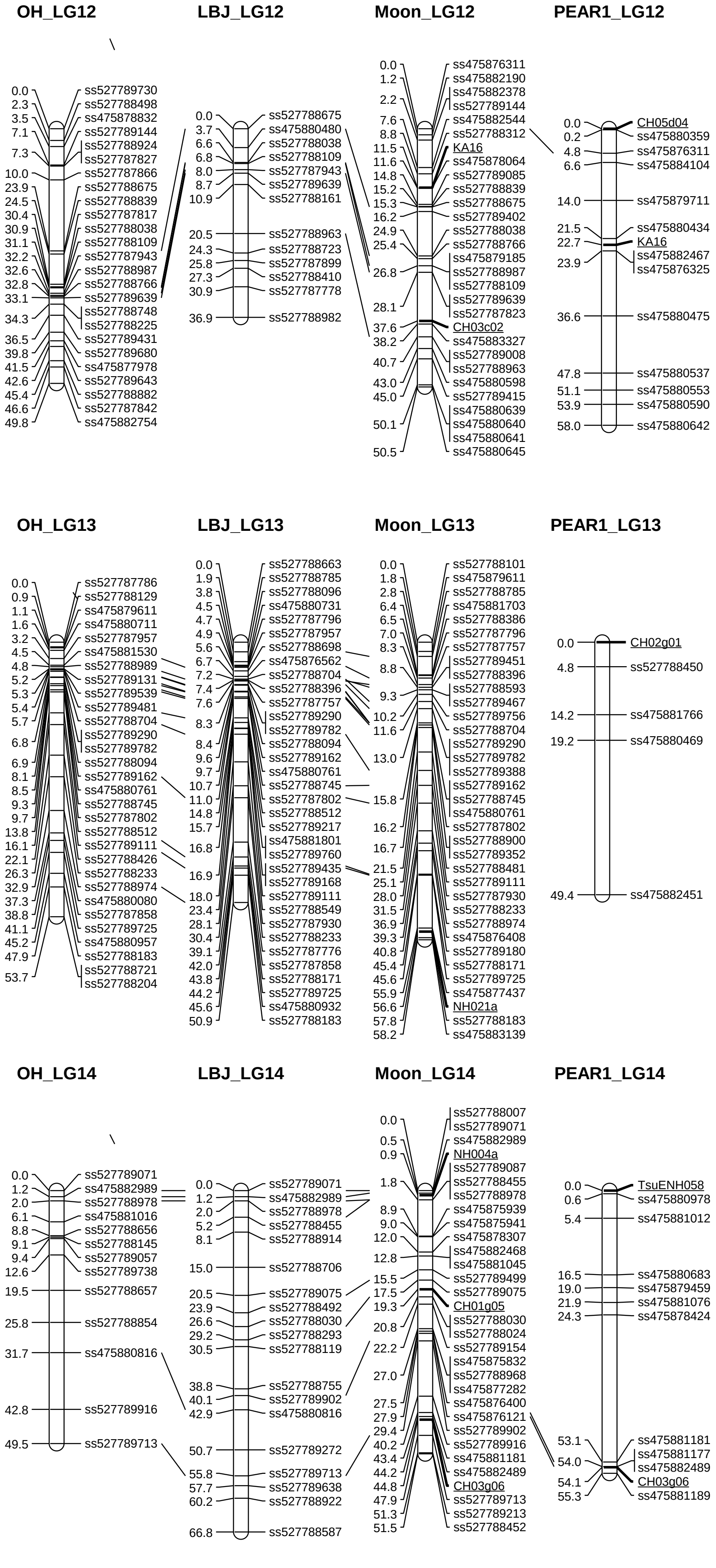

OH_LG12
0.0 2.3 3.5 7.1 7.3 10.0 23.9 24.5 30.4 30.9 31.1 32.2 32.6 32.8 33.1 34.3 36.5 39.8 41.5 42.6 45.4 46.6 49.8
ss527789730
ss527788498
ss475878832
ss527789144
ss527788924
ss527787827
ss527787866
ss527788675
ss527788839
ss527787817
ss527788038
ss527788109
ss527787943
ss527788987
ss527788766
ss527789639
ss527788748
ss527788225
ss527789431
ss527789680
ss475877978
ss527789643
ss527788882
ss527787842
ss475882754
LBJ_LG12
0.0 3.7 6.6 6.8 8.0 8.7 10.9 20.5 24.3 25.8 27.3 30.9 36.9
ss527788675
ss475880480
ss527788038
ss527788109
ss527787943
ss527789639
ss527788161
ss527788963
ss527788723
ss527787899
ss527788410
ss527787778
ss527788982
Moon_LG12
0.0 1.2 2.2 7.6 8.8 11.5 11.6 14.8 15.2 15.3 16.2 24.9 25.4 26.8 28.1 37.6 38.2 40.7 43.0 45.0 50.1 50.5
ss475876311
ss475882190
ss475882378
ss527789144
ss475882544
ss527788312
KA16
ss475878064
ss527789085
ss527788839
ss527788675
ss527789402
ss527788038
ss527788766
ss475879185
ss527788987
ss527788109
ss527789639
ss527787823
CH03c02
ss475883327
ss527789008
ss527788963
ss475880598
ss527789415
ss475880639
ss475880640
ss475880641
ss475880645
PEAR1_LG12
0.0 CH05d04
0.2 ss475880359
4.8 ss475876311
6.6 ss475884104
14.0 ss475879711
21.5 ss475880434
22.7 KA16
23.9 ss475882467
ss475876325
36.6 ss475880475
47.8 ss475880537
51.1 ss475880553
53.9 ss475880590
58.0 ss475880642
OH_LG13
0.0 0.9 1.1 1.6 3.2 4.5 4.8 5.2 5.3 5.4 5.7 6.8 6.9 8.1 8.5 9.3 9.7 13.8 16.1 22.1 26.3 32.9 37.3 38.8 41.1 45.2 47.9 53.7
ss527787786
ss527788129
ss475879611
ss475880711
ss527787957
ss475881530
ss527788989
ss527789131
ss527789539
ss527789481
ss527788704
ss527789290
ss527789782
ss527788094
ss527789162
ss475880761
ss527788745
ss527787802
ss527788512
ss527789111
ss527788426
ss527788233
ss527788974
ss475880080
ss527787858
ss527789725
ss475880957
ss527788183
ss527788721
ss527788204
LBJ_LG13
0.0 1.9 3.8 4.5 4.7 4.9 5.6 6.7 7.2 7.4 7.6 8.3 8.4 9.6 9.7 10.7 11.0 14.8 15.7 16.8 16.9 18.0 23.4 28.1 30.4 39.1 42.0 43.8 44.2 45.6 50.9
ss527788663
ss527788785
ss527788096
ss475880731
ss527787796
ss527787957
ss527788698
ss475876562
ss527788704
ss527788396
ss527787757
ss527789290
ss527789782
ss527788094
ss527789162
ss475880761
ss527788745
ss527787802
ss527788512
ss527789217
ss475881801
ss527789760
ss527789435
ss527789168
ss527789111
ss527788549
ss527787930
ss527788233
ss527787776
ss527787858
ss527788171
ss527789725
ss475880932
ss527788183
Moon_LG13
0.0 1.8 2.8 6.4 6.5 7.0 8.3 8.8 9.3 10.2 11.6 13.0 15.8 16.2 16.7 21.5 25.1 28.0 31.5 36.9 39.3 40.8 45.4 45.6 55.9 56.6 57.8 58.2
ss527788101
ss475879611
ss527788785
ss475881703
ss527788386
ss527787796
ss527787757
ss527789451
ss527788396
ss527788593
ss527789467
ss527789756
ss527788704
ss527789290
ss527789782
ss527789388
ss527789162
ss527788745
ss475880761
ss527787802
ss527788900
ss527789352
ss527788481
ss527789111
ss527787930
ss527788233
ss527788974
ss475876408
ss527789180
ss527788171
ss527789725
ss475877437
NH021a
ss527788183
ss475883139
PEAR1_LG13
0.0 CH02g01
4.8 ss527788450
14.2 ss475881766
19.2 ss475880469
49.4 ss475882451
OH_LG14
0.0 ss527789071
1.2 ss475882989
2.0 ss527788978
6.1 ss475881016
8.8 ss527788656
9.1 ss527788145
9.4 ss527789057
12.6 ss527789738
19.5 ss527788657
25.8 ss527788854
31.7 ss475880816
42.8 ss527789916
49.5 ss527789713
LBJ_LG14
0.0 ss527789071
1.2 ss475882989
2.0 ss527788978
5.2 ss527788455
8.1 ss527788914
15.0 ss527788706
20.5 ss527789075
23.9 ss527788492
26.6 ss527788030
29.2 ss527788293
30.5 ss527788119
38.8 ss527788755
40.1 ss527789902
42.9 ss475880816
50.7 ss527789272
55.8 ss527789713
57.7 ss527789638
60.2 ss527788922
66.8 ss527788587
Moon_LG14
0.0 0.5 0.9 1.8 8.9 9.0 12.0 12.8 15.5 17.5 19.3 20.8 22.2 27.0 27.5 27.9 29.4 40.2 43.4 44.2 44.8 47.9 51.3 51.5
ss527788007
ss527789071
ss475882989
NH004a
ss527789087
ss527788455
ss527788978
ss475875939
ss475875941
ss475878307
ss475882468
ss475881045
ss527789499
ss527789075
CH01g05
ss527788030
ss527788024
ss527789154
ss475875832
ss527788968
ss475877282
ss475876400
ss475876121
ss527789902
ss527789916
ss475881181
ss475882489
CH03g06
ss527789713
ss527789213
ss527788452
PEAR1_LG14
0.0 TsuENH058
0.6 ss475880978
5.4 ss475881012
16.5 ss475880683
19.0 ss475879459
21.9 ss475881076
24.3 ss475878424
53.1 ss475881181
54.0 ss475881177
ss475882489
54.1 CH03g06
55.3 ss475881189

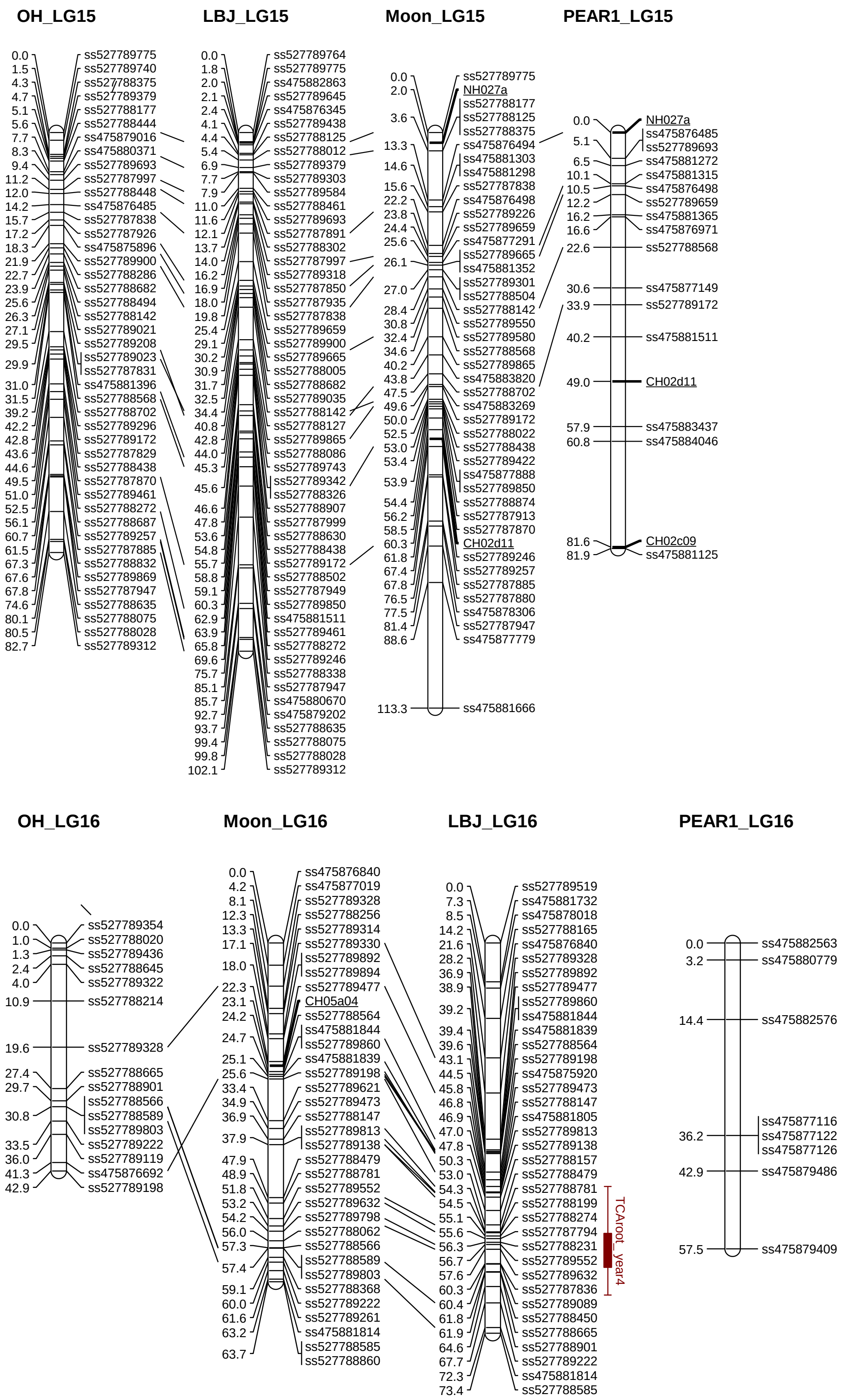
OH_LG15
0.0 1.5 4.3 4.7 5.1 5.6 7.7 8.3 9.4 11.2 12.0 14.2 15.7 17.2 18.3 21.9 22.7 23.9 25.6 26.3 27.1 29.5 29.9 31.0 31.5 39.2 42.2 42.8 43.6 44.6 49.5 51.0 52.5 56.1 60.7 61.5 67.3 67.6 67.8 74.6 80.1 80.5 82.7
ss527789775 ss527789740 ss527788375 ss527789379 ss527788177 ss527788444 ss475879016 ss475880371 ss527789693 ss527787997 ss527788448 ss475876485 ss527787838 ss527787926 ss475875896 ss527789900 ss527788286 ss527788682 ss527788494 ss527788142 ss527789021 ss527789208 ss527789023 ss527787831 ss475881396 ss527788568 ss527788702 ss527789296 ss527789172 ss527787829 ss527788438 ss527787870 ss527789461 ss527788272 ss527788687 ss527789257 ss527787885 ss527788832 ss527789869 ss527787947 ss527788635 ss527788075 ss527788028 ss527789312
LBJ_LG15
0.0 1.8 2.0 2.1 2.4 4.1 4.4 5.4 6.9 7.7 7.9 11.0 11.6 12.1 13.7 14.0 16.2 16.9 18.0 19.8 25.4 29.1 30.2 30.9 31.7 32.5 34.4 40.8 42.8 44.0 45.3 45.6 46.6 47.8 53.6 54.8 55.7 58.8 59.1 60.3 62.9 63.9 65.8 69.6 75.7 85.1 85.7 92.7 93.7 99.4 99.8 102.1
ss527789764 ss527789775 ss475882863 ss527789645 ss475876345 ss527789438 ss527788125 ss527788012 ss527789379 ss527789303 ss527789584 ss527788461 ss527789693 ss527787891 ss527788302 ss527787997 ss527789318 ss527787850 ss527787935 ss527787838 ss527789659 ss527789900 ss527789665 ss527788005 ss527788682 ss527789035 ss527788142 ss527788127 ss527789865 ss527788086 ss527789743 ss527789342 ss527788326 ss527788907 ss527787999 ss527788630 ss527788438 ss527789172 ss527788502 ss527787949 ss527789850 ss475881511 ss527789461 ss527788272 ss527789246 ss527788338 ss527787947 ss475880670 ss475879202 ss527788635 ss527788075 ss527788028 ss527789312
Moon_LG15
0.0 2.0 3.6 13.3 14.6 15.6 22.2 23.8 24.4 25.6 26.1 27.0 28.4 30.8 32.4 34.6 40.2 43.8 47.5 49.6 50.0 52.5 53.0 53.4 53.9 54.4 56.2 58.5 60.3 61.8 67.4 67.8 76.5 77.5 81.4 88.6 113.3
ss527789775 NH027a ss527788177 ss527788125 ss527788375 ss475876494 ss475881303 ss475881298 ss527787838 ss475876498 ss527789226 ss527789659 ss475877291 ss527789665 ss475881352 ss527789301 ss527788504 ss527788142 ss527789550 ss527789580 ss527788568 ss527789865 ss475883820 ss527788702 ss475883269 ss527789172 ss527788022 ss527788438 ss527789422 ss475877888 ss527789850 ss527788874 ss527787913 ss527787870 CH02d11 ss527789246 ss527789257 ss527787885 ss527787880 ss475878306 ss527787947 ss475877779 ss475881666
PEAR1_LG15
0.0 NH027a
5.1 ss475876485 ss527789693
6.5 ss475881272
10.1 ss475881315
10.5 ss475876498
12.2 ss527789659
16.2 ss475881365
16.6 ss475876971
22.6 ss527788568
30.6 ss475877149
33.9 ss527789172
40.2 ss475881511
49.0 CH02d11
57.9 ss475883437
60.8 ss475884046
81.6 CH02c09
81.9 ss475881125
OH_LG16
0.0 ss527789354
1.0 ss527788020
1.3 ss527789436
2.4 ss527788645
4.0 ss527789322
10.9 ss527788214
19.6 ss527789328
27.4 ss527788665
29.7 ss527788901
30.8 ss527788566 ss527788589 ss527789803
33.5 ss527789222
36.0 ss527789119
41.3 ss475876692
42.9 ss527789198
Moon_LG16
0.0 4.2 8.1 12.3 13.3 17.1 18.0 22.3 23.1 24.2 24.7 25.1 25.6 33.4 34.9 36.9 37.9 47.9 48.9 51.8 53.2 54.2 56.0 57.3 57.4 59.1 60.0 61.6 63.2 63.7
ss475876840 ss475877019 ss527789328 ss527788256 ss527789314 ss527789330 ss527789892 ss527789894 ss527789477 CH05a04 ss527788564 ss475881844 ss527789860 ss475881839 ss527789198 ss527789621 ss527789473 ss527788147 ss527789813 ss527789138 ss527788479 ss527788781 ss527789552 ss527789632 ss527789798 ss527788062 ss527788566 ss527788589 ss527789803 ss527788368 ss527789222 ss527789261 ss475881814 ss527788585 ss527788860
LBJ_LG16
0.0 7.3 8.5 14.2 21.6 28.2 36.9 38.9 39.2 39.4 39.6 43.1 44.5 45.8 46.8 46.9 47.0 47.8 50.3 53.0 54.3 54.5 55.1 55.6 56.3 56.7 57.6 60.3 60.4 61.8 61.9 64.6 67.7 72.3 73.4
ss527789519 ss475881732 ss475878018 ss527788165 ss475876840 ss527789328 ss527789892 ss527789477 ss527789860 ss475881844 ss475881839 ss527788564 ss527789198 ss475875920 ss527789473 ss527788147 ss475881805 ss527789813 ss527789138 ss527788157 ss527788479 ss527788781 ss527788199 ss527788274 ss527787794 ss527788231 ss527789552 ss527789632 ss527787836 ss527789089 ss527788450 ss527788665 ss527788901 ss527789222 ss475881814 ss527788585
TCAroot_year4
PEAR1_LG16
0.0 ss475882563
3.2 ss475880779
14.4 ss475882576
36.2 ss475877116 ss475877122 ss475877126
42.9 ss475879486
57.5 ss475879409

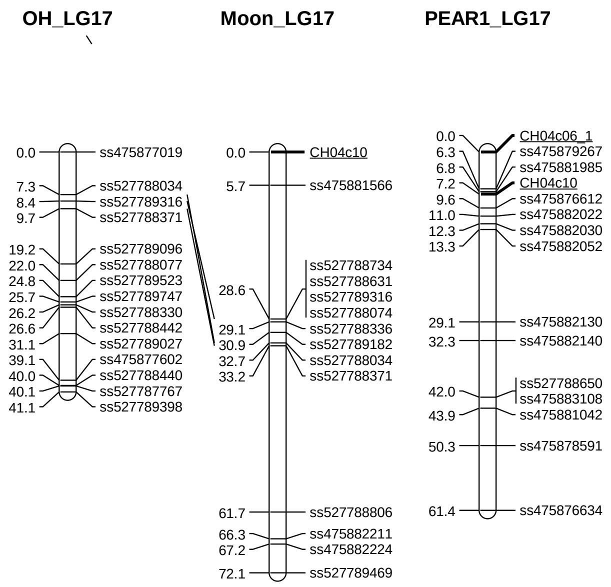
OH_LG17
Moon_LG17
PEAR1_LG17
0.0 ss475877019
7.3 ss527788034
8.4 ss527789316
9.7 ss527788371
19.2 ss527789096
22.0 ss527788077
24.8 ss527789523
25.7 ss527789747
26.2 ss527788330
26.6 ss527788442
31.1 ss527789027
39.1 ss475877602
40.0 ss527788440
40.1 ss527787767
41.1 ss527789398
0.0 CH04c10
5.7 ss475881566
28.6 ss527788734
ss527788631
ss527789316
ss527788074
29.1 ss527788336
30.9 ss527789182
32.7 ss527788034
33.2 ss527788371
61.7 ss527788806
66.3 ss475882211
67.2 ss475882224
72.1 ss527789469
0.0 CH04c06_1
6.3 ss475879267
6.8 ss475881985
7.2 CH04c10
9.6 ss475876612
11.0 ss475882022
12.3 ss475882030
13.3 ss475882052
29.1 ss475882130
32.3 ss475882140
42.0 ss527788650
ss475883108
43.9 ss475881042
50.3 ss475878591
61.4 ss475876634
